# Hereditary Patterns and Genetic Associations in Obsessive-Compulsive Disorder (OCD): Neuropsychiatric Insights, Genetic Influences, and Treatment Perspectives

**DOI:** 10.2174/0115665232316708240828063527

**Published:** 2024-08-30

**Authors:** Abhinay Dhiman, Sidharth Mehan, Zuber Khan, Aarti Tiwari, Ghanshyam Das Gupta, Acharan Singh Narula

**Affiliations:** 1 Division of Neuroscience, Department of Pharmacology, ISF College of Pharmacy, Moga, 142001, Punjab, India (Affiliated to IK Gujral Punjab Technical University, Jalandhar, Punjab, 144603), India;; 2 Department of Pharmaceutics, ISF College of Pharmacy, Moga, Punjab, India (Affiliated to IK Gujral Punjab Technical University, Jalandhar, Punjab, 144603), India;; 3 Narula Research, LLC, 107 Boulder Bluff, Chapel Hill, NC 27516, USA

**Keywords:** Obsessive-compulsive disorder, neuropsychiatry, epidemiology, genetics, diagnosis, comorbidity, treatment challenges

## Abstract

Obsessive-Compulsive Disorder (OCD), a prevalent neuropsychiatric condition, affects approximately 2%-3% of the global population. This paper provides an extensive overview of OCD, detailing its clinical manifestations, neurobiological underpinnings, and therapeutic approaches. It examines OCD's classification shift in the DSM-5, the role of the cortico-striato-thalamo-cortical pathway in its development, and the various factors contributing to its etiology, such as genes, environmental factors, and genetic predispositions. The challenges in diagnosing OCD and the effectiveness of both psychological and pharmacotherapeutic treatments are discussed. The paper also highlights the significant overlap between OCD and other mental health disorders, emphasizing its impact on global disability. Moreover, the role of genetic factors in OCD, including twin studies and gene association studies, is elaborated, underscoring the complex interplay of hereditary and environmental influences in its manifestation. The review further delves into the polygenic nature of OCD, illustrating how multiple genes contribute to its development, and explores the implications of genetic studies in understanding the disorder's complexity. Additionally, this research study delves into the concept of polygenic inheritance in complex diseases, highlighting the role of multiple genes in increasing OCD risk. A Genome-wide Association Study (GWAS) is employed to assess Single Nucleotide Polymorphisms (SNPs) to unearth genetic associations with OCD. This comprehensive analysis provides valuable insights into OCD's genetic landscape, paving the way for enhanced diagnostic approaches and treatment modalities.

## 1 INTRODUCTION

Obsessive-Compulsive Disorder (OCD) is a neuropsychiatric condition affecting 2%-3% of the global population [1]. It involves intrusive thoughts and repetitive behaviors, often accompanied by anxiety. OCD can manifest as compulsions, obsessions, or both, where individuals attempt to control these thoughts, impulses, or images by substituting alternative thoughts or actions [2, 3].

OCD is a disorder characterized by fear of contamination, obsessive handwashing, and concern about harm. It requires daily focus, affects daily life, and can impair performance. Previously classified as an anxiety disorder, OCD is now a distinct disorder within the category of Obsessive-compulsive and Related Disorders (OCRDs). However, OCD often co-occurs with other mental health disorders, highlighting the need for comprehensive treatment approaches [4].

The Cortico-stratum-thalmo-cortical pathway (CSTC) is crucial for OCD development, involving the prefrontal cortex, striatum, globus pallidus, and thalamus [5]. Dysfunction within the CSTC circuit, such as overactive striatum and disrupted communication, may contribute to OCD symptoms, including compulsive behaviors and difficulties in decision-making and emotional regulation [6, 7].

Significant contributors to the pathogenesis of OCD include environmental factors (childhood trauma, stress, socioeconomic status), neurotransmitter imbalances (serotonin, dopamine, glutamate), autoimmune mechanisms (PANDA), genetic predisposition, catecholaminergic genes (HTR1B, HTR2A, SLC6A4, COMT), and glutaminergic genes (GRIN2B, SLC1A1, SLITRK5, DLGAP2, DLGAP1). Furthermore, OCD has been linked to genes involved in cell growth and division (CTTNBP2), development and function of synapses (NRXN1), inactivation of catecholamine neurotransmitters (COMT, MAO-A, MAO-B), neurotransmitter synthesis alteration genes (TPH1 and TPH2), and numerous other gene types [8-11]. Along with this, various neuroimaging studies, including Magnetic Resonance Imaging (MRI) and its variants, like Voxel-based Morphometry (VBM), have highlighted brain abnormalities in OCD patients compared to healthy individuals [12]. These include reduced volumes in the Anterior Cingulate Cortex (ACC) and Orbitofrontal Cortex (OFC), areas involved in emotional regulation and decision-making. Functional MRI (fMRI) studies have shown increased activation in the Cortico-striato-thalamo-cortical (CSTC) pathway, related to habit formation and reward processing, potentially contributing to compulsive behaviors in OCD [13].

OCD often faces misdiagnosis, leading to delays in effective treatment due to challenges in clinical identification [14]. The Yale-Brown Obsessive-Compulsive Scale (Y-BOCS) is employed for severity evaluation. Early intervention with psychological techniques, like CBT, ERP, motivational interviewing, and pharmacotherapeutic approaches, has proven beneficial. Secondary pharmacological options include aripiprazole, risperidone, and clomipramine [15]. Adjunctive therapies, like deep-brain stimulation and neurosurgical ablation, have shown promise, especially for treatment-resistant OCD patients [16, 17].

## 2 PREVALENCE AND SIGNIFICANCE OF OCD WITH RESPECT TO MENTAL HEALTH

According to the National Institute of Mental Health, OCD is frequently accompanied by the concurrent presence of other mental health disorders, including panic disorder, generalized anxiety disorder, and depression [18]. OCD is one of the ten medical conditions that cause the most global disability, as reported by the WHO [19-22].

**Fig. (1):**
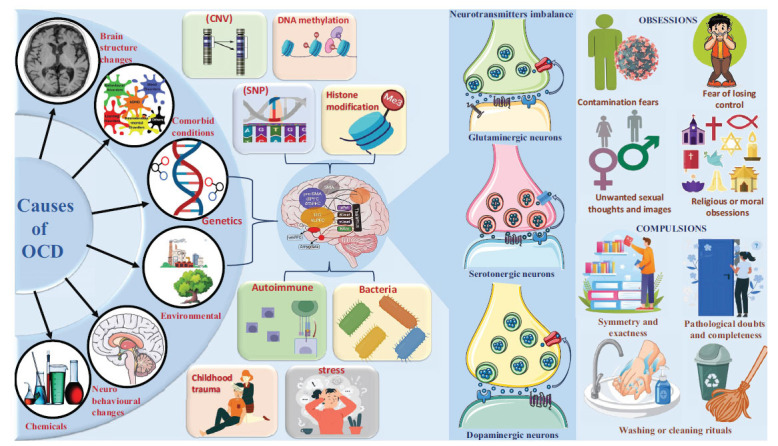
Multifaceted etiology of obsessive-compulsive disorder: neurobiological insight. In this figure, various causes of OCD have been depicted. Different causes of OCD include changes in brain structure, comorbid conditions, genetics, environmental factors, neurobehavioural changes, and various chemicals. Significant factors, like genetics and environmental elements, lead to most cases of OCD. Various environmental factors, such as autoimmune issues, bacteria, stress, and childhood trauma, along with genetic factors, like histone modification, single nucleotide polymorphism, copy number variation, and DNA methylation, lead to the dysfunction of the CSTC brain circuit. This dysfunction results in an imbalance of various neurotransmitters, like serotonin, dopamine, and glutamate. This disrupted balance of neurotransmitters causes various obsessive thoughts, such as contamination fears, fear of losing control, unwanted sexual thoughts and images, religious and moral obsessions, as well as various compulsive behaviors, like symmetry and exactness, pathological doubts and completeness, and washing or cleaning rituals. **Abbreviations:** CNV: Copy number variation; SNP: Single nucleotide polymorphism; OCD: Obsessive-compulsive disorder.

The prevalence of OCD in family members of individuals with early onset (≦18 years old) OCD is considerably higher than in families of individuals with later onset OCD [23-25]. The onset of OCD symptoms is subject to variation in age, with the majority of individuals manifesting symptoms during childhood around the age of ten. In contrast, others develop symptoms around the age of 21 during adolescence or young adulthood [26]. A meta-analysis review indicated the lifetime prevalence of OCD to be 1.5% for women and 1.0% for men, with women having a 1.6-fold increased risk compared to men [27, 28]. The prevalence of OCD in infants and adolescents is estimated to be between 1% and 3% [8]. OCD is a severe mental health condition that can significantly impair various aspects of an individual's life quality [29]. Social relationships constitute one domain that is notably affected by OCD [30]. Moreover, numerous epidemiological studies have linked OCD to an increased risk of suicidal behaviour (Fig. **1**).

## 3 GENETIC INFLUENCES ON OCD

### 3.1 Brief Explanation of Genetics and Heritability

Heredity and variation in living organisms are studied through molecular mechanisms involving genes, DNA, and genetics. Heritability is the proportion of trait variance within a population attributed to genetic influences, estimated using the formula H2 = Vg/Vp, where H represents heritability, Vg represents genotype variation, and Vp represents phenotypic trait variance [31].

Heritability, a measure of genetic influence on an attribute, is estimated using empirical data on familial resemblance [32]. It is typically determined through correlations between offspring and parental phenotypes, complete or half-sibling correlations, and differences between twin pairs [33].

OCD is familiar and heritable. Using univariate and multivariate twin models on a subset of twins (220 pairs), a twin study estimated the heritability of OC by TOCS and identified six OCD dimensions: symmetry/ordering, cleaning/contaminations, superstition, counting/checking, rumination, and hoarding. Regarding heritability, the total score of TOCS (74%) and each OC dimension (30-77%) was obtained [34]. A meta-analysis of previously analysed data revealed OCD as highly prevalent, particularly among children and adolescents; the heritability in twin samples was 0.5 [35]. An analysis of 18 OCD family studies revealed the prevalence of OCD in OCD families as approximately 7.2 times that of control families [36]. The author estimated the heritability of OCD at 29% using genetic variation from two homogeneous cohorts consisting of 4567 controls and 2090 Swedish-born individuals with OCD. The control group contained over 400000 single-nucleotide polymorphisms with a minor allele frequency of less than 0.01 [35].

### 3.2 Early Studies Suggesting Genetic Involvement in OCD

Numerous studies, including animal research, genetic linkage and association studies, twin research, and heritability estimates, have suggested the hereditary nature of OCD. OCD, like many other psychiatric disorders, is most likely the result of a complex interplay of inherited and environmental risk factors [33]. An extensive genetic component is implicated in OCD and related disorders, as supported by all twin and family aggregation studies [37]. The initial formal indication of genetic contribution occurred in 1965 when a case series revealed that monozygotic twins exhibited a higher concordance rate for OCD than dizygotic twins (22-47% *vs*. 70-80%). OCD was significantly more prevalent among first-degree relatives of adult OCD patients (ranging from 2.6% to 11.7%) and children with OCD (ranging from 5.0% to 22.7%), according to family study research. In contrast, the prevalence of OCD was lower among first-degree relatives of control subjects (ranging from 1.3% to 2.7% for adults and 0.0% to 0.9% for children) [38].

A recent meta-analysis revealed that additive genetic factors, which represent the influence of numerous genes (accounting for 37-41% of the variance), and non-shared environmental effects, such as unique life events experienced by one twin (accounting for 50-52% of the variance), are the primary contributors to the variation in obsessive-compulsive symptoms [38]. Non-additive genetic effects, such as epistatic or dominance effects, and shared environmental factors (*e.g*., parenting styles affecting both siblings) have negligible to no impact, contributing to only 5% to 6% and 9% to 10% variance, respectively [39].

A study on female twins found a positive correlation between obsession and compulsion symptoms and genetic factors contributing to compulsiveness. The study also found significant familial associations between sub-threshold OCD, tics, and anxiety disorders among 6-year-old twins, suggesting that genetic risk factors may play a role [40, 41].

Animal models, such as Global Sapap3-KO mice, have been reported to exhibit a pronounced phenotype. Sapap3, which is predominantly expressed in the striatum, neocortex, hippocampus, and thalamus, has been reported to be implicated in self-injurious grooming in mice (decreased mEPSC, fEPSP, and AMPA/NMDA ratio, and increased silent synapses and eCB-LTD). Knockout of this gene was accountable for mouse abnormalities [36, 42, 43].

A separate investigation involving Global (Slitrk5-KO) mice revealed that the knockout of the gene, accountable for self-injurious grooming in mice, impaired cortico-striatal function (reduced fEPSP), OFC hyperactivity (increased FosB staining levels), striatal volume, and MSN dendritic arbor complexity. Slitrk5 is primarily expressed in the striatum, neocortex, and hippocampus. Hoxb8, expressed in microglia derived from bone marrow and migrating to the OFC, cingulate cortex, and basal ganglia regions of the brain, is knocked out globally in mice with the pertinent phenotype [44, 45]. This gene regulates self-injurious grooming in mice [46, 47]. OCD has been linked genetically to several co-morbid conditions and disorders, including Tourette syndrome, Autism Spectrum Disorders (ASD), OCD, and Attention Deficit Hyperactivity Disorder (ADHD). Furthermore, OCD, ADHD, and anxiety disorders share genetic phenomenological characteristics, according to several studies [19-22, 41, 46, 47]. OCD's aetiology is supported by genetic evidence from animal studies, genetic linkage and association studies, twin studies, and genetic characteristics shared with other co-morbid conditions (GWAS).

### 3.3 Transition to the Polygenic Nature of OCD

In 1838, Jean-Étienne Esquirol was the first to document OCD in psychiatric literature. Lewis published one of the first family studies on OCD nearly a century later, in 1936. This research laid the foundation for the recognition of the significant role of heredity in the development of OCD [42]. Family-based studies serve as the foundation for genetic investigations into OCD. According to a 1964 study [21, 48-50], approximately 20 to 40% of first-degree relatives exhibit obsessive traits, and 4 to 8% of relatives of individuals with OCD also have OCD. After that, throughout the years, a multitude of twin studies have consistently underscored the considerable genetic influence on the etiology of OCD [20]. Consistently, these investigations have demonstrated OCD as hereditary, with those having a sibling, parent, or child with the disorder being at an increased risk. Their research on OCD symptom dimensions [48, 49] has discovered hoarding and contamination/cleaning symptoms to be more well-known than others. A different study found familial OCD as associated with a higher prevalence of symmetry-related symptoms than sporadic cases [50]. Family studies have demonstrated their utility in the investigation of OCD-related disorders across a spectrum. Major depressive disorder, agoraphobia, panic disorder, generalized anxiety disorder, and separation anxiety disorder have all been reported as more prevalent among case relatives compared to the control group [51, 52]. Grooming disorders (nail biting, skin plucking, trichotillomania) and body dysmorphic disorder have also been reported to be more prevalent in case relatives than in the control group in the research [51, 52]. Numerous subsequent twin studies have demonstrated the heritability of OCD [49]. Prior research has focused on individual genes; candidate gene studies, on the other hand, have identified particular genes by analyzing their variations in relation to the observed phenotype and established a connection between those variations and the pathogenesis of the disease [38, 53].

Recent research on Obsessive-Compulsive Disorder (OCD) suggests a polygenic hypothesis involving multiple genes in its development. Genome-wide association studies have identified potential SNPs and genes associated with OCD's pathophysiology. OCD is now recognized as a heritable polygenic disorder [41].

## 4 POLYGENIC INHERITANCE

### 4.1 Explanation of Polygenic Inheritance

Polygenic inheritance is a genetic pattern where multiple genes impact a trait, resulting in a continuous, quantitative phenotype. This phenomenon is common in complex diseases and is observed in traits, like height, circadian rhythms, obesity, diabetes, schizophrenia, and bipolar disorder [46].

### 4.2 Contribution of Multiple Genes to Complex Traits

Polygenic inheritance, involving multiple genes influencing traits, is common in complex diseases, like schizophrenia, bipolar disorder, obesity, diabetes, and mental health disorders. Genome-wide association studies have identified single nucleotide polymorphisms as associated with these traits, highlighting their polygenic nature [54].

Alternatively referred to as quantitative or polygenic traits, complex traits manifest as a continuous spectrum of variation rather than straightforward Mendelian inheritance patterns. Multiple genes and environmental factors impact these traits, including height, intelligence, and blood pressure. These traits are frequently evaluated using quantitative methods and are not governed by a solitary gene through simple dominant or recessive inheritance. GWASs have identified over 600 variants correlated with human height, with early studies pinpointing SNPs at 47 loci and subsequent meta-analyses linking thousands of SNPs to biological pathways influencing adult height. Similarly, blood pressure is a complex trait with 30-50% heritability, according to GWAS, which has associated 280 genetic variants with increased hypertension risk, also implicated in coronary artery disease [46, 55]. Essential genes regulating circadian rhythms include the Cry, Per, Bmal1, Clock, Npas2, Dec, Rora, Dbp, Reverb, and E4bp4 families [56, 57]. The research has identified additional genes associated with cognitive functioning and temperament, underscoring the polygenic nature of these traits [58].

### 4.3 Traits Associated with OCD Control by Various Genes

Various features of OCD are controlled by different genes, such as BDNF, CREB, SLC6A4, SLC1A1, and GRIN2B genes [55], which govern memory. The genes SLC1A1, DRD2, and DRD1 control compulsions, which are repetitive behaviours and mental activities. The genes SLC6A4 and DRD2 govern motivation and reward functions. The HTR1B genes influence feeding behaviour, while the DRD2 genes regulate drinking habits. Lastly, the SLC6A3 genes control locomotory activity. This illustrates the impact of gene numbers on complex features.

### 4.4 Polygenic Basis of Various Medical and Psychological Conditions

#### 4.4.1 Medicinal Illnesses

**Fig. (2):**
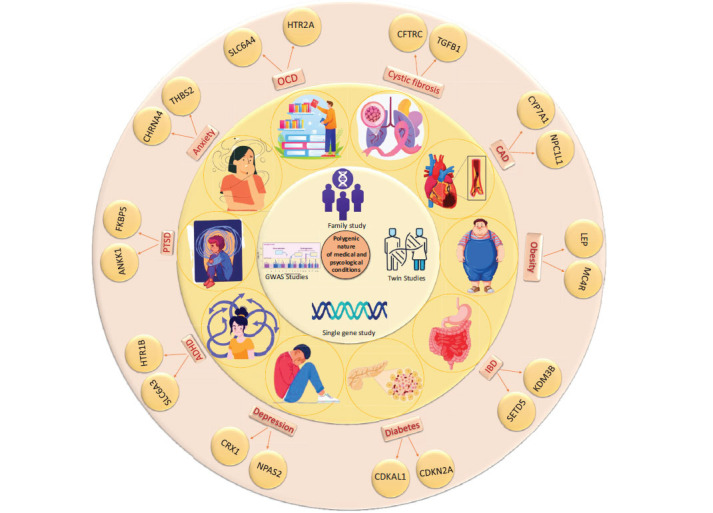
Genetic correlates of medical and psychological conditions: insights from diverse studies. This diagram presents various studies that aid in identifying genes associated with different medical and psychological conditions. These studies encompass family, twin, single-gene, and GWAS. Different types of genes linked to various medical and psychological conditions are discerned through these investigations. The genes include SLC6A4 and HTRA2, associated with OCD; CFTRC and TGFB1, linked to cystic fibrosis; CYP7A1 and NPC1L1, related to CAD; LEP and MC4R, connected to obesity; SETD5 and KDM3B, implicated in IBD; CDKAL1 and CDKN2A, involved in diabetes; CRX1 and NPAS2, associated with depression; SLC6A3 and HTR1B, linked to ADHD; ANKK1 and FKBP5, connected with PTSD; CHRNA4 and THBS, associated with anxiety. All these genes, connected with various medical conditions, are identified through these diverse studies. **Abbreviations:** SLC6A4: Solute carrier family 6 member 4 (dopamine transporter); HTRA2: High-temperature requirement protein 2; CFTRC: Cystic fibrosis transmembrane conductance regulator; TGFB1: Transforming growth factor beta 1; CYP7A1: Cytochrome P450, family 7, subfamily A, polypeptide 1 (cholesterol 7-alpha-hydroxylase); NPC1L1: Niemann-Pick disease, type C1, protein like 1; LEP: Leptin; MC4R: Melanocortin 4 receptor; SETD5: SET domain-containing 5; KDM3B: Lysine (K)-specific demethylase 3B; CDKAL1: CDKAL1 cadherin-related family member 1; CDKN2A: Cyclin-dependent kinase inhibitor 2A (p16INK4a); CRX1: Cone-rod homeobox protein; NPAS2: Neuronal PAS domain protein 2; SLC6A3: Solute carrier family 6 member 3 (dopamine transporter); HTR1B: 5-hydroxytryptamine receptor 1B; ANKK1: Angiogenesis regulator with SH3 domain protein 1; FKBP5: FK506 binding protein 5; CHRNA4: Cholinergic receptor, nicotinic, alpha 4 (neuronal); THBS: Thrombospondin 1; GWAS: Genome-wide association study; OCD: Obsessive-compulsive disorder; CAD: Coronary artery disease; IBD: Inflammatory bowel disease; ADHD: Attention deficit hyperactivity disorder; PTSD: Post-traumatic stress disorder.

Obsessive-Compulsive Disorder (OCD) shares several polygenic risk factors with ADHD, autism, schizophrenia, Bipolar Disorder (BD), and Major Depressive Disorder (MDD). Key genes involved in the progression of OCD include CACNA1C, ANK3, DRD1, DRD5, SLC18A1, and HTR2, with the SLC and DRD gene families playing significant roles. Notably, DRD5, SLC18A1, and HTR2 are also implicated in BD, which share genetic loci, such as ANK3 and CACNA1C, with schizophrenia, affecting amygdalar activity and neuronal discharge [59, 60]. Schizophrenia and OCD share common genes, including SLC6A4, BDNF, HTR2A, and DRD2. ADHD is linked to serotonin transporter and receptor genes SLC6A4, SLC6A3, and HTR1B, which are also involved in other neurodevelopmental disorders [61]. Furthermore, OCD is genetically associated with PTSD, MDD, and anxiety through genes, such as ANKK1, FKBP5, CHRNA4, and THBS2. Mutations in SETD5 and KDM3B connect OCD with Inflammatory Bowel Disease (IBD), underscoring the polygenic nature and genetic interconnections of these conditions [62] (Fig. **2**).

## 5 UNRAVELING THE POLYGENIC NATURE OF OCD

### 5.1 Genetic Studies and Twin Studies Highlighting Genetic Factors

Twin research and genetic studies have significantly contributed to understanding the impact of genetic factors on human characteristics and diseases. Sequencing studies, such as whole genome sequencing and GWAS, help estimate genetic contributions to complex traits, like OCD. OCD is heritable, with females having 18% heritability and males having 23% [48].

A further analysis of 21911 pairs of twins determined the genetic heritability to be 45% [63]. In addition to this, numerous twin studies have demonstrated OCD as heritable genetically [34, 61]. In addition, some family-based studies, including one involving 1224 OCD subjects and 52 OCD-affected multigenerational families, have determined a genetic heritability of 52%. An additional study involving 3,494 OCD patients from 1,365 families determined a heritability of 37% [19, 20]. A GWAS study involving 4236 controls and 1061 OCD cases determined heritability to be 37%, and another GWAS study involving 7037 controls and 2688 OCD cases determined heritability to be 28% (IOCDF-GC; International Obsessive Compulsive Disorder Foundation-Genetics Collaborative). Furthermore, numerous other GWAS studies have demonstrated OCD to be heritable genetically [64, 65]. Lin *et al.* identified four genes, SETD5, KDM3B, ASXL3, and FBL, through whole-genome sequencing on 53 parent-offspring families, indicating chromatin modification's role in OCD development, confirming its polygenic nature.

### 5.2 Advances in the Technology and Genomic Research

Mutations in OCD are polygenic and can be investigated using various analytical techniques. The human genome has been mapped for genetic testing, identifying disease-associated gene variants. Methods include whole exome sequencing, noninvasive prenatal testing, and next-generation sequencing. Whole genome sequencing and bioinformatics are used to identify OCD-associated variants. Advancements in next-generation sequencing and panel assays are used in noninvasive prenatal testing. Precision medicine requires this information for identifying gene variants.

## 6 GENOME-WIDE ASSOCIATION STUDY (GWAS) ON OCD

### 6.1 Explanation of GWAS

GWAS is a method used to identify genomic variants linked to a specific trait or disease risk by analyzing the genomes of numerous individuals [66]. This technique identifies more prevalent variants among those with the disease or trait, allowing researchers to investigate adjacent variants [67].

Genome-wide Association Studies (GWAS) compare allele frequencies using SNPs in genomic risk loci to identify significant trait associations within similar individuals with divergent phenotypes. The process involves study design, data collection, quality control, imputation, association testing, meta-analysis, replication, interpretation, publication, and follow-up research. DNA is extracted from blood or cell samples and analyzed for specific genetic markers. A GWA study in 2007 identified numerous genetic polymorphisms associated with seven diseases.

### 6.2 Major Findings and Identified Genetic Markers

The variants of medical and neurological conditions are investigated using GWAS. The initial successful Genome-wide Association Study (GWAS) on myocardial infection was published in 2002. It identified an SNP in intron 1 of LTA, encoding lymphotoxin-α (252A→G) on chromosome 6p21, linked to myocardial infarction [58, 68]. The GWA 2005 study, which examined 96 cases and 50 controls for polymorphisms associated with age-related macular degeneration, identified two variants in the Complement Factor H (CFH) locus as strongly associated with AMD: rs380390 and rs10272438 [69]. Diverse genomic risk loci have been linked to diseases and traits, including PTPN22 for autoimmune disease and FTO (Fat mass and Obesity-associated) for obesity, according to a decade of GWAS [70]. Occasionally, these results provide insights into the biology of diseases. Including the IL-12/IL-23 pathway has been linked to the advancement of Chron's disease [71].

GWAS has identified 44 genetic markers linked to disease progression in major depressive disorder, including SNP rs1432639, rs2303222, and rs2075650. These variants have been reported to affect mitochondrial trafficking, neuronal growth, synaptic transmission, and plasticity, contributing to conditions, like autism, bipolar disorder, and schizophrenia [72].

A meta-analysis identified six variants linked to Alzheimer's disease, ADHD, and autism. Intronic variants near the APP gene, missense variants near the SHARPIN gene, and a variant near the CHRNE gene were associated with decreased risk. A meta-analysis of 12 studies found 12 significant loci at the genome level, with the FOXP2 gene associated with ADHD and the MACROD2 gene linked to autism. These variants have been reported to be predominantly expressed in the lungs and brain regions [73].

Variants near the NEGR1 locus on chromosome 1, such as rs1620977, are linked to ASD, MDD, and schizophrenia. NEGR1 regulates synapse formation and neurite outgrowth in hippocampus neurons. ASD is also associated with variant rs201910565 near the PTBP2 gene on chromosome 1, which regulates alternative splicing, and with variant rs1452075 near the CADPS gene on chromosome 3, involved in neurotransmitter exocytosis and cognitive decline.

A meta-analysis of ADHD, schizophrenia, ASD, MDD, and BPD identified common variants across these conditions. Variant rs2535629 near the ITIH3 gene on chromosome 3, which regulates neuronal plasticity, is implicated in all five disorders. Additionally, variants rs11191454 and rs2799573 near AS3MT and CACNB2 genes, and rs1024582 near CACNA1C on chromosome 10, are linked to these disorders. The CACNA1C gene, crucial for Ca^2+^ ion transport in the brain and heart, is involved in memory, anxiety response, and rapid nerve signal transmission.

### 6.3 Significance of these Markers in Understanding OCD

The genes' SNP, or single nucleotide polymorphism, serves as the main indicator in GWAS investigations of different neurological illnesses. This phenomenon of polymorphism results in a multitude of different variations that are involved in the development of the disease. Various Single Nucleotide Polymorphisms (SNPs) have been implicated in neuropsychiatric illnesses, such as autism, ADHD, schizophrenia, bipolar disorder, and major depressive disorder. This indicates that Single Nucleotide Polymorphism (SNP) variants may also influence Obsessive-Compulsive Disorder (OCD).

## 7 GENETIC VARIANTS AND BIOLOGICAL PATHWAYS

Numerous genetic variants are linked to OCD, affecting neurotransmitter types and receptors in the caudate nucleus, OFC, anterior thalamus, and ACC, responsible for sensory processing, decision-making, and habit formation [38].

### 7.1 Specific Genetic Variants Associated with OCD

Numerous studies, including family-based, genome-wide association, and twin research, have established the genetic component of OCD. One study identified that the G allele at rs6662980 in SAPAP3 influences OCD by affecting glutamate and dopamine signaling in the striatum, which contributes to compulsive washing behaviors through heightened threat processing, dysfunctional reward processing, and enhanced habit formation [63, 72]. There is a possibility that BDNF and its specific receptor, Neurotrophic Tyrosine Kinase Receptor type 2 (NTRK2), contribute to an increased susceptibility to OCD by affecting the functions of BDNF, such as neuroregeneration, synaptic plasticity, serotonin regularization, and inflammatory and immune response [43, 74]. A statistically significant correlation (*p* <.0001) was observed between an NTRK2 intronic SNP (rs2378672) and OCD among female patients. A study analysed the data to examine the association between OCD and SNP Val66Met (rs6265) at position 66 in the coding exon of the BDNF gene. A lack of statistically significant association was observed between the rs6265 polymorphism and OCD, as determined by analysis. However, a gender-specific analysis suggested that female Val carriers may pose an OCD risk factor [75].

Six additional BDNF variants (rs3763965, rs2352802, rs972096, rs1387145, rs1464896, and rs2140887) have been identified in one study as being linked to OCD [76]. A single study demonstrated that the neuronal cadherin gene (CDH2) contributes to the onset of OCD under the additive allelic model, and the single nucleotide polymorphism rs12605662 is significantly associated with OCD (*p* < 0.001), with each G-allele addition considerably reducing the likelihood of OCD in individuals. These variations likely influence OCD risk by enhancing brain circuit development, promoting proper glutamate signaling, and supporting healthy synapse formation.

SNPs rs11930311, rs4617664, rs7938406, rs3785931, rs7649709, and rs1526083 are associated with OCD, respectively, according to one study. These SNPs are near the GLRB, GRIP1, NCAM1, NGFR, NLGN1, and PIK3CG genes. These polymorphisms potentially influence OCD risk by affecting nearby genes critical for glutamatergic signaling (GRIP1, GLRB), neuronal communication and plasticity (NCAM1, NGFR), synapse formation (NLGN1), and cellular signaling pathways (PIK3CG), potentially leading to circuit abnormalities relevant to OCD. Three SNP markers for OLIG2 on chromosome 21q were identified: rs762178 (minor allele frequency: 35%; *P*<.001); rs9653711 (minor allele frequency: 44%; *P* =.004); and rs1059004 (minor allele frequency: 44%; *P* =.005). These markers are associated with OCD. The OLIG2 gene is highly expressed in brain regions involved in remyelination and neurogenesis, including the amygdala, thalamus, and caudate nucleus, crucial for white matter cell development. Thus, variation in the gene leads to demyelination, and a decrease in neuronal circuit connectivity leads to OCD [75, 76]. OCD is associated with rodents lacking the BTBD3 gene. A trio analysis identified the SNP rs6131295 on chromosome 20p12.1-p12.2, approximately 90 kb from the BTBD3 gene, with a *p*-value of 3.84×10^-8, indicating genome-wide significance. BTBD3 is involved in various cellular processes, including protein ubiquitination and degradation, transcriptional regulation, cytoskeleton dynamics, and ion channel assembly and gating.

An exon-focused genome-wide association study identified a shared polygenic risk for OCD and schizophrenia at SNP rs12151009 on chromosome 19:46141845, near the EML2 gene. This SNP may contribute to OCD susceptibility by affecting microtubules, synaptic function, and immune-related pathways [43, 68, 77]. A two-sample Mendelian Randomization (MR) study identified SNP rs5757717 near CACNA1I, associated with both Schizophrenia (SCZ) and Obsessive-Compulsive Disorder (OCD) (FDR = 2.12 × 10^−2^). Variations at this locus disrupt calcium channels, affecting neurotransmitter release and neuronal firing, thereby increasing the risk for both SCZ and OCD [78].

A GWAS study using the TOCS on 5,018 Caucasian children and adolescents identified a significant association at SNP rs7856850 within the PTPRD gene. Polygenic risk scores and meta-analysis of case/control datasets linked this locus to OC traits (*p* = 2.48 × 10^−8^) and OCD (*p* = 0.0069). Variations in PTPRD are associated with glutamate receptor dysregulation, altering brain circuits, and decreasing communication between brain regions, which is linked to obsessions and compulsions [32].

### 7.2 Involvement of Neurotransmitter Systems (Serotonin, Dopamine, Glutamate)

Serotonin, a monoamine in the gastrointestinal tract, blood platelets, and central nervous system, regulates mood, emotion, pleasure, memory, cognition, anxiety, appetite, stress, addiction, and sleep [79].

Tryptophan, the precursor to serotonin, undergoes hydroxylation to form 5-HTP and decarboxylation to yield serotonin (5-HT). The crucial enzyme, Tryptophan Hydroxylase type 2 (TPH2), located in 5-HT neurons, catalyzes the rate-limiting conversion of tryptophan to 5-HTP. Serotonin is mainly released from medullary nuclei and influences stress, depression, and anxiety through its extensive network of ascending and descending serotonergic neurons, particularly from the forebrain-projecting raphe nuclei (DRN and MRN) characterized by various techniques, such as immunohistochemistry, viral genetics, and electrophysiology [80]. The 5-HT system in rodents can regenerate after neurotoxin or brain injury [80, 81]. Serotonin, binding to 14 receptors in the brain, has diverse functions, with the 5HT-3 receptor being a unique ligand-gated ion channel [73, 82, 83]. Anxiety behavior is linked to 5-HT neurons co-expressing vesicular glutamate transporter-3, thus impacting glutamate neurotransmission [84].

#### 7.2.1 Other Effects of Serotonin

Ocular effects: Serotonin can increase intraocular pressure and induce pupil dilation by stimulating ciliary body muscle fibers [85]. The inotropic and chronotropic effects of serotonin benefit the cardiovascular system, as it causes tachyarrhythmias by increasing intracellular calcium in cardiac myocytes [86]. Platelet granules contain serotonin, which affects platelet aggregation and causes vasodilation in healthy endothelium and vasoconstriction in the damaged endothelium [87]. Serotonin regulates uterine muscle tone, micturition, oocyte maturation, penile detumescence, and uterine vasoconstriction in the genitourinary system [88].

Metabolic and endocrine effects: Serotonin promotes liver lipid accumulation, regulates pancreatic secretion, and enhances insulin release and glucose uptake in muscles. Additionally, it stimulates adipose tissue lipogenesis [89]. Serotonin facilitates gastric emptying, improves intestinal motility, stimulates intestinal secretion, and enhances colonic tone in the gastrointestinal tract [89].

#### 7.2.2 5-HT-1 Receptors

The 5-HT1 receptor family, distinguished by its intron less coding sequence, is the largest class of 5-HT receptors, consisting of five subtypes that share 40-63% sequence homology [77]. Except for the 5-HT3 receptor, which is ionotropic, all 5-HT receptors are G Protein-coupled Receptors (GPCRs). 5-HT receptors, which bind to distinct subunits of GPCR (subunit names), exhibit excitatory and inhibitory effects. Among the 5-HT1Rs, those associated with Gαi/Gαo proteins include 5-HT1AR, 5-HT1BR, 5-HT1DR, 5-HT1ER, and 5-HT1FR. On the other hand, 5-HT2Rs, including 5-HT2AR, 5-HT2BR, and 5-HT2C, stimulate Gαq proteins. Lastly, 5-HT4R, 5-HT6R, and 5-HT7R are linked to Gαs proteins [90]. Diverse brain regions contain 5-HT1A, 1B, 1D, 1E, and 1F receptors, each with pharmacological properties.

5-HT1A receptors are G protein-coupled receptors that exhibit inhibitory activity when coupled to a G protein. The activation of 5-HT1A receptors in the brain results in hyperpolarization of the postsynaptic neurons and decreased firing rate. Early research development found 5-HT1A receptors to be extensively dispersed throughout the cerebellum, substantia nigra, limbic regions, raphe nuclei (where they function as autoreceptors on 5-HT neurons), and extrapyramidal areas, including the caudate-putamen and substantia nigra [77, 90-93]. Astrocytic 5-HT1A receptors facilitate neuroprotective processes, such as inhibiting oxidative stress and inflammation. Acquiring knowledge and cognition are facilitated by 5-HT1A receptors [94-96]. Initial results obtained from a variety of investigations employing distinct cell systems, neuronal cell lines, neurons, and *in vivo* experiments indicated that the 5-HT1A receptor functions as a Gi/o-coupled receptor, wherein signals are transmitted *via* the conventional pathway of Adenylyl Cyclase (AC) inhibition. Neuronal activity is diminished when the 5-HT1A receptor is coupled with Gβγ subunits, which facilitate the opening of potassium channels and the closing of calcium channels [97]. The activity of Extracellula-regulated protein Kinase (ERK) ERK1/2 is modulated by the 5-HT1A receptor, which exerts an inhibitory and activating effect on synaptogenesis and the behavioural effects of antidepressants. Recent studies have indicated that it exerts its antidepressant effects, activates ACII, PLC/PKC, CAMKII, and PI3K/Akt signalling pathways, and interacts with Gβγ and tyrosine kinase receptors [98-101].

Inhibition of adenylyl cyclase by a 5-HT1B agonist decreases the production of cyclic Adenosine Monophosphate (cAMP). This crucial second messenger plays a role in ion channel regulation, metabolism, and gene expression. cAMP is also implicated in aggression, melancholy, and anxiety.

The striatum, frontal cortex, basal ganglia, and hippocampus have higher concentrations of 5HT1D receptors, which modulate brain functions, like cognition, mood, and behavior by inhibiting neurotransmitters. Serotonin binding to these receptors reduces cyclic AMP levels, affecting protein synthesis, gene expression, and ion channel activity. 5-HT1E receptors in the frontal cortex and hippocampus may regulate memory. Recent cloning of the 5-HT(1F) receptor and its affinity for sumatriptan could revolutionize anti-migraine therapies [102, 103].

#### 7.2.3 5-HT2

The 5-HT2 receptor subtypes (5-HT2A-C) share molecular structure, pharmacology, signal transduction similarities, and an amino acid sequence similarity of approximately 50%. Cortical regions (entorhinal, neocortex, and piriform cortex), the olfactory tubercle, dentate gyrus, numerous brainstem nuclei, motor cranial nerve nuclei, and the ventral horn of the spinal cord all express the 5-HT2A receptor [102, 103].

All 5-HT2 receptor subtypes (5-HT2A-C) are Gq-mediated G Protein-coupled Receptors (GPCRs) that stimulate phospholipase C and protein kinase C, which are involved in additional brain functions. Hallucinations, anxiety, depression, aggression, reward, sensory prediction, and pain perception are all correlated with the 5-HT2A receptor [104-106].

The serotonin 2A receptor (5-HT2AR) plays complex roles in behaviour and cognition. In rodents, 5-HT2AR agonism increases impulsivity, while its role in human aggression is unclear. Mice lacking 5-HT2AR showed reduced anxiety, normalizing upon receptor restoration [107]. Activation of 5-HT2AR improves cognition, particularly learning and neurogenesis in cortical regions, with agonists, like psilocybin and LSD, enhancing cognitive flexibility and creative thinking [108-111].

The serotonin 2B receptor (5-HT2BR), expressed in various human tissues [112-115], functions as a Gq/11 protein-coupled receptor, activating signaling pathways, such as PLCβ and PKC. It modulates serotonin-related cognitive processes, including memory and learning, and its regulation of dopamine release suggests therapeutic potential for neuropsychiatric disorders, like schizophrenia [116, 117].

Serotonin 2C receptors (5-HT2CRs), predominantly found in the limbic system, cortex, and basal ganglia, regulate nociception, motor behaviour, endocrine secretion, and appetite. They are crucial for reward-based learning and behaviours relevant to psychiatric disorders, with therapeutic targeting explored for conditions, like obesity, schizophrenia, anxiety, depression, Parkinson's disease, and substance addiction.

#### 7.2.4 5HT3

5HT3 receptors are distinct from other serotonin receptors in that they function *via* Ligand-gated Ion Channels (LGICs) instead of the G proteins. Five subunits are symmetrically arranged around a central ion-conducting pore to form the 5-HT3 receptor [118]. Two subunits, 5-HT3A and 5-HT3B, have been identified in rats and rodents; these subunits are capable of forming both homomeric 5-HT3A receptors and heteromeric 5-HT3A/5-HT3B receptors [118, 119]. There are three further subunits, specifically 5-HT3C, 5-HT3D, and 5-HT3E, which have been identified in several mammalian species, but not in rodents [120, 121].

Brain regions, such as the cortex, hippocampus, nucleus accumbens, substantia nigra, and ventral tegmental area, contain 5-HT3 receptors, which are involved in cognitive processes, anxiety, and emesis. The brainstem regions associated with the vomiting reflex, including the area postrema and nucleus tractus solitaries, contain the highest concentrations of 5-HT3 receptors [122]. These receptors, located in both presynaptic and postsynaptic nerve terminals, facilitate the release of numerous neurotransmitters, including dopamine, GABA, and cholecystokinin, by stimulating a rapid increase in cytosolic calcium *via* calcium influx [123-127]. These receptors are involved in the vomiting reflex in the brainstem, anxiety mediation in the amygdala and hippocampus, and rapid excitatory synaptic transmission in the neocortical interneurons and ferret visual cortex. Interneurons containing 5HT3 receptors regulate the release of glutamate and GABA, thereby playing a role in various neuropsychiatric disorders.

#### 7.2.5 5-HT4

The 5HT4 receptor is a Gs protein-coupled receptor that increases cellular cyclic Adenosine Monophosphate (cAMP) levels and stimulates adenylyl cyclase [128]. 5HT4 receptors are present in every cell of the body. Peripheral components include the gastrointestinal tract, the heart, and the urinary bladder, and they perform various functions. 5HT4 receptors are predominantly localized in the basal ganglia and limbic structures, integral to cognition, and implicated in many pathological disorders [128]. According to one study, 5-HT4R KO mice exhibited no discernible variations in body weight, metabolism, sleep pattern, or social behaviour [129]. When Ach is released in the frontal cortex and hippocampus, 5HT4 induces an excitatory response linked to memory and cognition [130].

5-HT4 receptors, Gs protein-coupled receptors, modulate dopamine and GABA release, influencing synaptic plasticity and cognitive functions [89, 121]. In the globus pallidus, serotonin-dependent 5-HT4 receptor activation increases GABA release and neuronal firing *via* presynaptic and postsynaptic mechanisms. The 5-HT4 receptor agonist BIMU-8 indirectly enhances GABA release in the hippocampus through cholinergic muscarinic receptors [123]. Subchronic 5-HT4 receptor agonist treatment induces biochemical and behavioural changes in the hippocampus akin to prolonged SSRI treatment, including CREB phosphorylation and neurogenesis [131, 132]. 5-HT4 receptors also facilitate dopamine release in the striatum, a process inhibited by receptor antagonists [133]. Systemic 5-HT4 receptor stimulation or overexpression in the mPFC increases DRN neuron firing, indicating a positive feedback loop between the PFC and DRN, involving cortical 5-HT4 receptor activation, glutamate release in the DRN, and increased DRN firing [131-133]. Furthermore, these receptors are implicated in memory, anxiety, depression, Alzheimer's disease, schizophrenia, ADHD, and Parkinson's disease [132, 134, 135].

#### 7.2.6 5-HT5R

The central and peripheral nervous systems both contain the 5-HT5 receptor family, which consists of two members: 5-HT(5)A and 5-HT(5)B. The 5-HT(5)A receptor is present in humans, mice, and rats, whereas the 5-HT(5)B receptor is non-existent in mice, but detectable in rats [136]. The 5-HT(5A) receptor exhibits a broad distribution throughout the central nervous system, while the 5-HT(5B) receptor is confined to a restricted number of sites. By coupling with G proteins, primarily *via* Gi/o, 5-HT5A receptors inhibit adenylyl cyclase activity. In addition to motor control, learning, and memory consolidation, anxiety and depression are also influenced by this receptor.

#### 7.2.7 5-HT6R

Initially cloned from striatal tissue, the 5-HT6 receptor is the most recently identified member of the serotonin (5-HT) receptor superfamily. Primarily found in the Central Nervous System (CNS), including the amygdala, hippocampus, and cerebral cortex, the 5-HT6R is involved in memory and learning [18]. The distribution of 5-HT6 mRNA in the central nervous system of rats was determined through *in situ* hybridization and northern blot analyses. The olfactory tubercle contained the highest concentration, followed by the frontal and entorhinal cortices, and dorsal hippocampus (including dentate gyrus and CA1, CA2, and CA3 regions, nucleus accumbens, and striatum) [137]. Several diencephalic nuclei, including the hypothalamus, amygdala, and substantia nigra, exhibited decreased expression levels. In conjunction with immunolocalization and radioligand investigations, the distribution of 5-HT6 receptor protein in the central nervous system of rats was also monitored [138]. Several behavioural parameters, including social recognition (MWM) [137, 138], Novel Object Recognition (NOR), and non-spatial visual learning and memory (NOR), indicate that 5HT6R is involved in cognition (social cognition and NOR, respectively).

#### 7.2.8 5-HT7R

One of the most recent additions to the serotonin receptor family is the 5-HT7 receptor. Agonists and antagonists, as well as 5-HT7R knock-out rodents, demonstrate the involvement of this receptor in processes of the central nervous system [135]. G-protein-coupled receptor 5-HT7R interacts with Gα12 proteins, which are involved in neurite outgrowth and retraction. It stimulates adenylate cyclase *via* Gαs proteins, thereby increasing cAMP [139, 140]. The regions containing the most significant number of 5-HT7 receptors are the dentate gyrus and the anterior thalamus. Specific brainstem nuclei, including the hypothalamus, anterior cingulate gyrus, hippocampus, and amygdala, contain moderate 5-HT7 receptors [141]. The 5-HT7 receptor has been implicated in a variety of *in vivo* studies utilising diverse animal models to examine depression, sleep, circadian rhythms, anxiety, OCD, cognition, schizophrenia, epilepsy, cognition, and migration, among other diseases and disorders [135].

### 7.3 Serotonin-related Genetic Variants that Cause OCD

#### 7.3.1 5HT1

Blood samples from 205 patients and 207 controls were genotyped in a PCR-RFLP study. Statistically speaking, only the rs10042486 CT genotype among HTR1A polymorphisms was associated with dysregulation of serotonin and associated with OCD symptoms [135].

#### 7.3.2 5HT2

Adults with OCD have a positive association with the A allele of the 5-HT2A receptor promoter polymorphism 1438G/A, according to one study. Furthermore, a study identified significant differences in both allele frequencies (*P* < 0.05) and genotype (*P* < 0.05) between the control group and individuals with OCD [142]. Genotyping analysis was performed on candidate serotonin genes (SLC6A4, HTR2A, HTR1B, and HTR2C), and structural Magnetic Resonance Imaging (sMRI) was employed to determine regional brain volumes within CSTC circuits in a study involving 200 paediatric subjects. The researchers investigated the correlation between serotonin gene variants and OCD, in addition to the effects of these variants on brain volume in males and females individually, irrespective of diagnostic status. Gender differences in diagnosis and genotype were found to be statistically significant for two HTR2C SNPs: rs12860460 (estimated interaction of 5.45 cc, *P* value 9.70e-8) and rs12854485 (estimated interaction of 4.28 cc, *P* value 2.07e-6), but not in males [143]. Using a transmission-disequilibrium test, a 54–parent-child trios study identified a significant association between rs6311 at the HTR2A gene and OCD patients. This gene is accountable for modulating the mood, thoughts, and behaviour of those with OCD [144].

#### 7.3.3 5HT3

A significant correlation between the HTR3C variant p.N163K (rs6766410) and OCD was identified in male patients, with genotype (*p*=0.027) and allele effects (*p*=0.007) indicating a higher prevalence of the c.489A-allele encoding the p.163K variant in male OCD patients compared to controls (49% *vs*. 37%; OR=1.61, CI=1.15-2.26). The HTR3E gene variant p.T86A (rs7627615) was notably associated with the “washing” subtype of OCD and visual impairment, as well as cognitive organization in unaffected relatives and controls [145]. The HTR3D variant rs1000592 (p.H52R) demonstrated a nominally significant association with OCD in case-control analysis (*p*=0.029), which remained significant in combined case-control and trio analyses (*p*=0.024).

In a comparison of 599 controls and 596 OCD individuals, a significant genotypic distribution difference was observed for the HTR3B variant rs1176744, with an Odds Ratio (OR) of 0.74 (CI=0.60–0.91, *P*=0.0043). Additionally, an HTR3B protective haplotype was associated with OCD (OR=0.77, CI=0.63–0.95, permuted *P*=0.0179). Significant associations were also found between rs3758987 and early-onset OCD in males (OR=0.49, CI=0.31–0.79, *P*=0.0031), and between rs6766410 and rs6443930 near HTR3C and HTR3D genes and the cleaning dimension in females (OR=0.36, CI=0.18–0.69, *P*=0.0016; OR=0.47, CI=0.29–0.79, *P*=0.0030, respectively).

#### 7.3.4 5HT4-7

The evidence regarding a direct association between variants of genes associated with 5HT4-7 and OCD is currently inconclusive and preliminary. Additional investigation is required to elucidate their function and possible therapeutic ramifications [141].

## 8 VNTR POLYMORPHISM

**Fig. (3):**
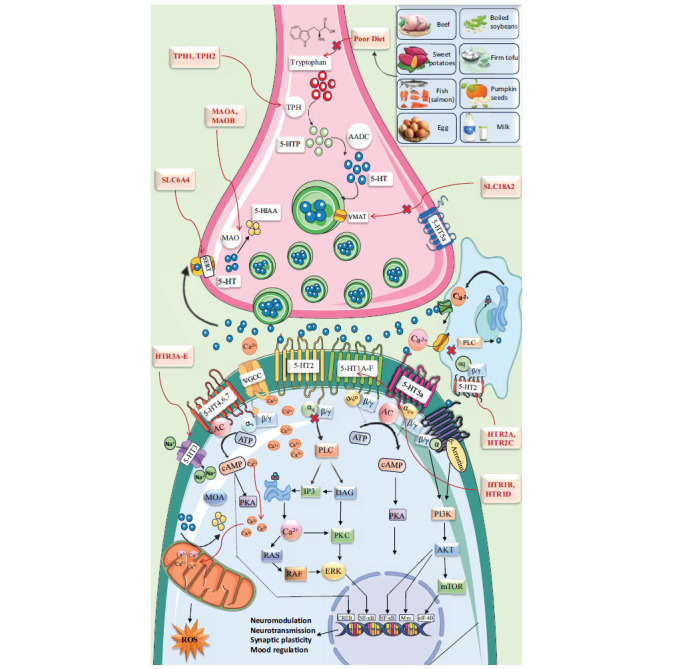
Genetic pathways implicated in OCD: serotonergic dysregulation and receptor mutations. In this diagram, various gene mutations associated with OCD have been mentioned. A poor diet leads to lower production of tryptophan, ultimately resulting in decreased production of 5-HT and, thereby, dysregulation of the serotonergic pathway. Mutations in the TPH1 and TPH2 genes inhibit TPH, which is responsible for converting tryptophan to 5-HTP, causing a reduction in 5-HT levels. Mutations in the MAOA and MAOB genes interact with MAO, increasing the level of 5-HIAA, the degraded product of 5-HT, leading to further decreases in 5-HT. The SERT receptor, involved in 5-HT reuptake, contributes to OCD pathogenesis. The mutated SLC6A4 gene increases 5-HT reuptake by the SERT receptor, decreasing 5-HT levels in the synaptic cleft. The VMAT receptor, facilitating 5-HT transfer into synaptic vesicles, is blocked due to a mutation in SLC18A2, causing a decrease in synaptic cleft 5-HT levels. Gene mutations, like HTR3A to E, interact with the 5-HT3 receptor, which generally transports Na+, K+, and Ca2+ into neurons. Mutations in these genes impede this transport function. Low 5-HT levels in the synaptic cleft increase intracellular calcium levels in neurons, causing mitochondrial dysfunction by elevating ROS levels. Disturbances in receptors 5-HT5a and 5-HT1A to 5-HT1F are associated with OCD pathogenesis *via* cAMP dysregulation. Mutations in HTR1B and HTR1D genes are linked to OCD, causing an increase in NF-κB in the nucleus. Dysregulation of 5-HT4, 6, and 7 increases CREB levels in the nucleus, affecting various processes, like neuromodulation, neurotransmission, synaptic plasticity, and mood regulation. Mutations in HTR2A and HTR2C interfere with the 5-HT2 receptor present in glial cells. β-arrestin, associated with a GPCR receptor, contributes to OCD pathogenesis by elevating PI3K, AKT, and mTOR levels. This elevation increases Myc, elf-4B, and NF-κB levels in the nucleus, impacting neuromodulation, neurotransmission, synaptic plasticity, and mood regulation. **Abbreviations:** TPH: Tryptophan hydroxylase; MAO: Monoamine oxidase; SLC6A4: Solute carrier family 6, member 4 (serotonin transporter); 5-HTP: 5-hydroxytryptophan; AADC: Aromatic L-amino acid decarboxylase; VMAT: Vesicular monoamine transporter; 5-HIAA: 5-hydroxyindoleacetic acid; SLC18A2: Solute carrier family 18, member 2 (vesicular monoamine transporter 2); HTR3: 5-hydroxytryptamine receptor 3; PLC: Phospholipase C ; AC: Adenylyl cyclase; ATP: Adenosine triphosphate; cAMP: Cyclic adenosine monophosphate; PKA: Protein kinase A; RAS: Rat sarcoma viral oncogene homolog ; RAF: Rapidly accelerated fibrosarcoma; ERK: Extracellular signal-regulated kinase; PKC: Protein kinase C ; IP3: Inositol trisphosphate; DAG: Diacylglycerol ; PI3K: Phosphatidylinositol 3-kinase; mTOR: Mammalian target of rapamycin; AKT: Protein kinase B ; CREB: cAMP response element-binding protein ; NF-κB: Nuclear factor-kappa B; elF-4B: Eukaryotic initiation factor 4B ; Myc: Myc proto-oncogene protein ; ROS: Reactive oxygen species. *Symbols: 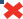 inhibition*.

A comparative study identified a VNTR polymorphism in intron 2 of the serotonin transporter (5-HTT) gene involving Stin2.9, Stin2.10, and Stin2.12 alleles. This variant has been associated with a range of psychiatric disorders, including unipolar depression, bipolar disorder, schizophrenia, and anxiety disorders, including OCD [141] (Fig. **3**).

## 9 ROLE OF DOPAMINE ASSOCIATED WITH OCD

Dopamine, a monoamine/catecholamine neurotransmitter, functions in the central and peripheral nervous systems. It is synthesized in the substantia nigra, hypothalamus, and ventral tegmental area [146]. Dopamine regulates motor functions, motivation, reward, arousal, euphoria, lactation, sexual behaviour, nausea, and the sleep-wake cycle [69]. It also facilitates synaptic plasticity in the striatum and prefrontal cortex, influencing cognition, motivation, emotion, and neuroendocrine secretion [147-149].

Dopamine biosynthesis begins with the amino acid tyrosine, converted to L-DOPA by tyrosine hydroxylase in dopaminergic neurons [150]. Dopamine is metabolized by Monoamine Oxidase (MAO) and Catechol-O-methyl Transferase (COMT) into inactive metabolites. MAO-A, primarily found in catecholaminergic neurons, and MAO-B, located mainly in astrocytes, degrade dopamine into 3,4-dihydroxyphenylacetic acid (DOPAC) and Homovanillic Acid (HVA) [151].

Dopaminergic neurons are located in the substantia nigra pars compacta, hypothalamus, and midbrain, projecting through mesocortical, mesolimbic, and nigrostriatal pathways to various brain regions. They exert effects *via* five subtypes of G protein-coupled receptors: D1, D2, D3, D4, and D5. The receptor density ranks in the human central nervous system [152].

## 10 DOPAMINE RECEPTORS

Dopamine receptors are essential in various brain regions, impacting physiological and behavioural functions [153]. D1 receptors in various brain regions regulate attention, learning, memory, sleep, impulse control, and renal function by regulating adenylate cyclase [154]. D2 receptors in the striatum, ventricle-talone, olfactory bulb, and cerebral cortex are primarily inhibitory, influencing post-synaptic and pre-synaptic functions. D3 receptors, D4 receptors, and D5 receptors regulate sleep, attention, impulse control, and cognition. Their distinct distributions contribute to the complex regulation of neurobiological processes [155, 156].

## 11 GENETIC VARIANTS ASSOCIATED WITH THE DOPAMINERGIC SYSTEM

A study examining the relationship between OCD and high/low activity polymorphisms of Catechol-O-methyltransferase (COMT) NlaIII and dopamine D(2) receptor (DRD2) TaqI A found no significant differences between controls and OCD patients. However, male OCD patients had a higher frequency of DRD2 A2 alleles and a correlation with early-onset OCD. A Ser9Gly polymorphism in the DRD3 gene was associated with an increased risk of OCD diagnosis, with the male gender being a significant predictor [156].

Gene-gene associations of MAOA, MAOB, and COMT gene variants, including independent polymorphisms in the MAOB gene (rs6651806 and rs1799836) and rs362204 (COMT), which are associated with an increased risk of OCD, were reported in a pilot study [157].

An investigation was conducted on a 48-base pair polymorphism situated in the third exon of the Dopamine Receptor type 4 (DRD4) gene. The study identified a significant correlation between DRD4 gene variants, specifically allele 2, and OCD across population and family-based studies. This indicates that allele two or an adjacent genetic variation might protect against the symptoms of OCD [158].

The risk of OCD was reported to be significantly associated with the COMT Val158Met polymorphism in a meta-analysis of 14 case-control studies involving a total of 2753 healthy controls and 1435 OCD cases. Males exhibited the strongest association, indicating that the COMT Val158Met polymorphism is a substantial risk factor for OCD, at least among this gender. The COMT Val158Met (rs4680; 472G -> A) was identified as the specific polymorphism associated with OCD risk, especially in males. The results of a case-control study involving 327 healthy subjects and 87 adults with OCD indicated a genetic association between the met(158) allele of catechol-O-methyltransferase (COMT) and OCD, particularly in men. This association was also observed in previous cases, but not in females [159-161].

**Fig. (4):**
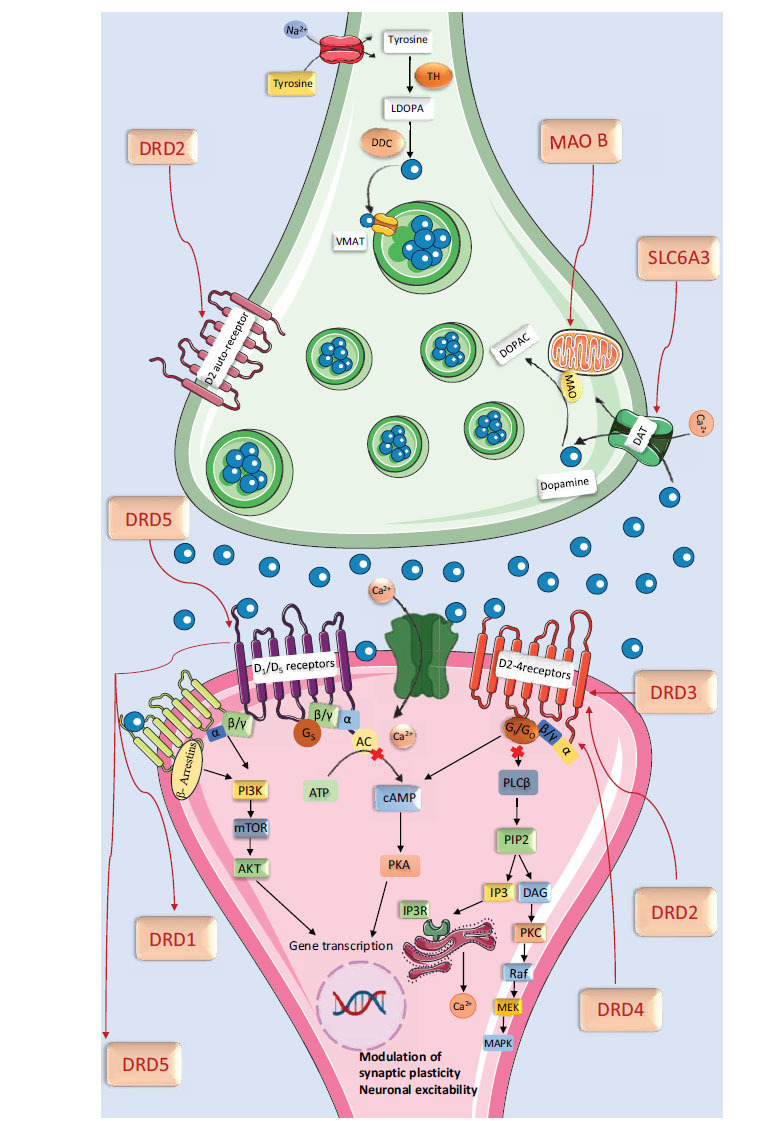
Gene mutations and dopaminergic pathway dysregulation in OCD progression. This diagram illustrates various gene mutations associated with the dopaminergic pathway. The D2 receptor plays a crucial role in both the synthesis and signaling of dopamine. However, mutations in the DRD2 gene disrupt the fundamental functions of the D2 autoreceptor, contributing to the pathogenesis of OCD. Similarly, mutations in the DRD5 gene result in functional defects in the D1/D5 receptors, preventing the conversion of ATP to cAMP in adenyl cyclase. Consequently, this leads to a decrease in the levels of PKA, causing some transcriptional defects. Functional defects in the D1/D5 receptor also stem from mutations in the DRD1 gene. Additionally, mutations in the DRD2, DRD3, and DRD4 genes are responsible for the dysregulation of the D2, D3, and D4 receptors, respectively. These receptors are pivotal in the signaling of the IP3 and DAG pathways. However, gene mutations disrupt these signaling pathways, contributing to the pathogenesis of OCD. Mutations in the SLC6A3 receptor alter the modulation of the DAT transporter, impacting dopamine levels in the presynaptic neuron. Similarly, mutations in the MAOB gene also affect dopamine levels in the presynaptic neuron. These mutations across these genes lead to defects in synaptic plasticity and neuronal excitability, contributing to the progression of OCD. **Abbreviations:** TH: Tyrosine hydroxylase; L-DOPA: L-3,4-dihydroxyphenylalanine; DDC: DOPA decarboxylase; VMAT: Vesicular monoamine transporter; DOPAC: 3,4-dihydroxyphenylacetic acid; MAO: Monoamine oxidase; DAT: Dopamine transporter; SLC6A3: Solute carrier family 6, member 3 (dopamine transporter); DRD2: Dopamine receptor D2; PI3K: Phosphatidylinositol 3-kinase; mTOR: Mammalian target of rapamycin; AKT: Protein kinase B; ATP: Adenosine triphosphate; cAMP: Cyclic adenosine monophosphate; PKA: Protein kinase A; IP3R: Inositol trisphosphate receptor; AC: Adenylyl cyclase; PLCβ: Phospholipase C beta; PIP2: Phosphatidylinositol bisphosphate; IP3: Inositol trisphosphate; DAG: Diacylglycerol; PKC: Protein kinase C; Raf: Rapidly accelerated fibrosarcoma; MAPK: Mitogen-activated protein kinase kinase. *Symbols: 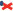 inhibition*.

Evidence from the study's examination of the COMT Val (158)met polymorphism's effect on COMT enzyme activity and its participation in cortical dopamine signalling supports the notion that it might contribute to the genetic underpinnings of OCD, particularly in males. Additionally, the results emphasize the existence of sexual dimorphism in both COMT and OCD. OCD was found to have a significant association (p0.00019) with the DAT3 gene polymorphism (rs3773679) in 54 child and adolescent OCD patients, according to one study [162]. Polymorphisms in various receptors, transporters, and proteins lead to dysregulation of dopamine levels in the brain, contributing to compulsive actions, repetitive behaviors, and reward-seeking activities in OCD (Fig. **4**).

## 12 ROLE OF GLUTAMATE IN OCD

Glutamate, classified as a non-essential amino acid, is synthesized through various metabolic pathways [163]. In glial cells, glutamine is a primary precursor, which is converted to glutamate by glutamate phosphate-activated glutaminase within mitochondria, yielding glutamate and ammonia. Lysine and other amino acids also contribute to glutamate synthesis *via* decarboxylation and deamination processes [164]. Glutamate can also be derived from glucose through intermediates of the Tricarboxylic Acid (TCA) cycle. It is stored in presynaptic vesicles and released through a calcium-dependent exocytosis mechanism, leading to rapid accumulation at high concentrations [165-167]. Extracellular glutamate levels are regulated by non-vesicular release *via* the cystine-glutamate antiporter, with imbalances potentially causing energy depletion or cell death. As the most abundant neurotransmitter in the brain, glutamate functions as an excitatory neurotransmitter, playing a critical role in synaptic plasticity, immune functions, and behavioural processes, such as learning and memory, by interacting with various receptor types [168, 169].

Glutamate binds primarily to two receptor families: ionotropic (iGluR) and metabotropic (mGluR) receptors. Ionotropic receptors, which include kainate, NMDA, and AMPA receptors, function as voltage-gated ion channels. Metabotropic receptors are G Protein-coupled Receptors (GPCRs) with seven transmembrane domains that initiate signaling cascades upon glutamate binding [168, 169]. These mGluRs are further classified into three groups: group 1 (mGluR1, mGluR5), which includes excitatory members and activates phospholipase C, increasing inositol triphosphate and calcium levels; and groups 2 (mGluR2, mGluR3) and 3 (mGluR4, mGluR6, mGluR7, mGluR8), which are inhibitory and reduce intracellular cAMP by inhibiting adenylyl cyclase [168, 170, 171].

Extracellular glutamate levels in the CNS are regulated by sodium-dependent Excitatory Amino Acid Transporters (EAATs) of the Solute Carrier family 1 (SLC1) [208, 209], present in neurons and astrocytes. These transporters ensure proper synaptic function by managing extracellular glutamate levels. Additionally, Vesicular Glutamate Transporters (VGLUTs 1-3) and the glutamate/aspartate transporter carry glutamate for synaptic release [168, 172].

## 13 METABOTROPIC AND IONOTROPIC RECEPTORS WITH ENCODED GENES

Metabotropic receptors of groups I, II, and III, including mGluR1, mGluR2, mGluR3, mGluR4, mGluR5, mGluR7, and mGluR8, are found in the cerebral cortex, hippocampus, and cerebellum, playing crucial roles in memory and learning by regulating synaptic plasticity. mGluR6, located in the retina, regulates glutaminergic neurotransmission in bipolar cells. These receptors are encoded by various genes: GRM1 and GRM5 for group I (mGluR1, mGluR5), GRM2 and GRM3 for group II (mGluR2, mGluR3), and GRM4, GRM6, GRM7, and GRM8 for group III (mGluR4, mGluR6, mGluR7, mGluR8) [172]. Ionotropic receptors, or excitatory amino acid receptors, include NMDA, AMPA, and kainate receptors. These receptors enhance learning and memory by inducing Long-term Potentiation (LTP) and increasing synaptic plasticity. The NMDA receptor has seven subunits (GluN1, GluN2a-d, GluN3a-b), AMPA has four subunits (Glu1-Glu4), and kainate has four subunits (GluK1-GluK4). Orphan receptors δ1 and δ2 share similarities with iGluR. Specific genes associated with these subunits include GRIN1 (GluN1), GRIN2A-D (GluN2A-D), and GRIK1-4 (GluK1-4) [173-175].

## 14 GLUTAMATE TRANSPORTERS AND ENCODED GENES

Vesicular Glutamate Transporter (VGLUT) and its subtypes are also present, in addition to the diverse Excitatory Amino Acid Transporter (EAAT) and its subtypes (EAAT1-5).

VGLUT1-3 is a vesicular glutamate transporter found in the brain that regulates the expression of numerous genes. Genes SLC1A3, SLC1A2, SLC1A1, and SLC1A6 encode the transporters EAAT1, EAAT2, EAAT3, and EAAT4, respectively; SLC17A6 and SLC17A8 encode the transporters VGLUT2 and VGLUT3 [43].

## 15 GLUTAMATE GENETIC VARIANTS ASSOCIATED WITH OCD

Numerous studies have demonstrated the involvement of glutaminergic genes in OCD. A twin and family-based study implicated SLC1A1 gene variants in OCD. SLC1A1 encodes the EAAC1 glutamate transporter, essential for clearing the excitatory neurotransmitter glutamate from the synaptic space. Variants rs301435 and rs301434 may impair EAAC1 function, leading to elevated glutamate levels and excitotoxicity, affecting brain development and plasticity. Significant associations with OCD were observed for rs301435 (χ^2^ = 9.24; *P* = 0.03) and rs301434 (χ^2^ = 12.04; *P* = 0.006) on chromosome 9p24 [176].

An additional family-based analysis was conducted, which included 71 probands and their parents who had been diagnosed with OCD according to the DSM-III-R or DSM-IV. At nine distinct positions within the SLC1A1 gene region, SNPs were genotyped. The SLC1A1 gene region was found to have significant associations with OCD, specifically at rs3780412 (*P* =.04) and rs301430 (*P* =.03), with the latter demonstrating effects being gender-specific and exclusive to males. Furthermore, a T/C haplotype located at rs301430-rs301979 exhibited a significant association (*P* =.03), with male probands comprising the majority once more [177].

A total of 662 controls, matched by ethnicity and sex, and 325 OCD probands participated in a case-control study to determine the role of genetic variation in SLC1A1, the gene responsible for glutamate transport, in OCD. A family-based study of 47 OCD probands and their parents analyzed the GRIK2 gene's association with OCD using Haploview and FBAT software. The SNP rs1556995 is significantly associated with OCD (*P* = 0.0027). GRIK2 encodes glutamate receptors, and its polymorphism disturbs glutamate levels in brain circuits. Additionally, haplotypes rs1556995 and rs1417182 were significantly associated with OCD.

Comparative research reveals that OCD and Anorexia Nervosa (AN) share several genetic variants. Neurogenesis-related variants, including rs4825476 (GRIA3) with a *p*-value of 4 × 10^−4^ and rs11783752 (SCL18A1) with a *p*-value of 3 × 10^−3^, were associated with both AN and OCD. During a case-control association study, ten tag SNPs were genotyped in the 3'-untranslated Region (3' UTR) of GRIN2B encoding for the NR2 subunit of NMDA glutamate receptors. A total of 279 control participants and 225 OCD patients were recruited from the OCD Clinic at Bellvitge Hospital in Barcelona, Spain. The examination of OCD sub-phenotypes revealed the rs1805476 Single Nucleotide Polymorphism (SNP) as significantly correlated (*p* = 0.002) with obsessions regarding contamination and cleaning in male patients (95% Confidence Interval (CI): 1.37-4.22).

Using real-time polymerase chain reaction, researchers investigated the impact of the GRIN2B polymorphism (rs1019385) in a cohort comprising healthy controls and Brazilian Obsessive-Compulsive Disorder (OCD) patients. They found a significant association between the TT genotype and the T-allele with OCD, particularly in dimensions related to ordering and checking behaviors. This association may be attributed to increased glutamate levels mediated by the NMDA glutamate receptor [178]. This study aimed to examine the potential correlation between genetic variations linked to the onset of OCD and the concentrations of diverse neurometabolites in the Anterior Cingulate Cortex (ACC). Neurometabolic levels were concurrently assessed using 3-T (1)H-MRS, and 262 Single Nucleotide Polymorphisms (SNPs) in 35 genes were investigated in a cohort of 41 paediatric OCD patients. The analysis identified significant associations between variants in the vesicular monoamine transporter 1 gene (SLC18A1, rs6586896) and the ionotropic AMPA1 gene (GRIA1, rs2963944 and rs707176) with OCD. These variants affect serotonin and dopamine neurotransmitter storage in synaptic vesicles and elevate glutamate levels, respectively, contributing to OCD pathophysiology [179].

Family studies, twin studies, and segregation analysis conducted on 356 control individuals revealed the association of three novel variants with seven individuals; individuals with OCD were examined for missense N400I mutations and it was found that N400I variants failed to promote neurite outgrowth in primary neuronal cultures when compared to wild-type SLITRK1. Additionally, one individual with OCD exhibited a synonymous L63L genetic change, while four individuals with OCD possessed an additional missense change, T418S, which was linked to OCD. The genotyping of 203 patients and 203 control samples was performed utilising the TaqMan^®^ method in a study. A statistically significant association was observed between the SLITRK1 variant (rs9593835) and checking dimensions, particularly among male patients (*P* = 0.04), which may suggest a potential link to OCD [179, 180].

## 16 GENES ASSOCIATED WITH THE SYNTHESIS, STORAGE, RELEASE, AND METABOLISM OF NEUROTRANSMITTERS

**Fig. (5):**
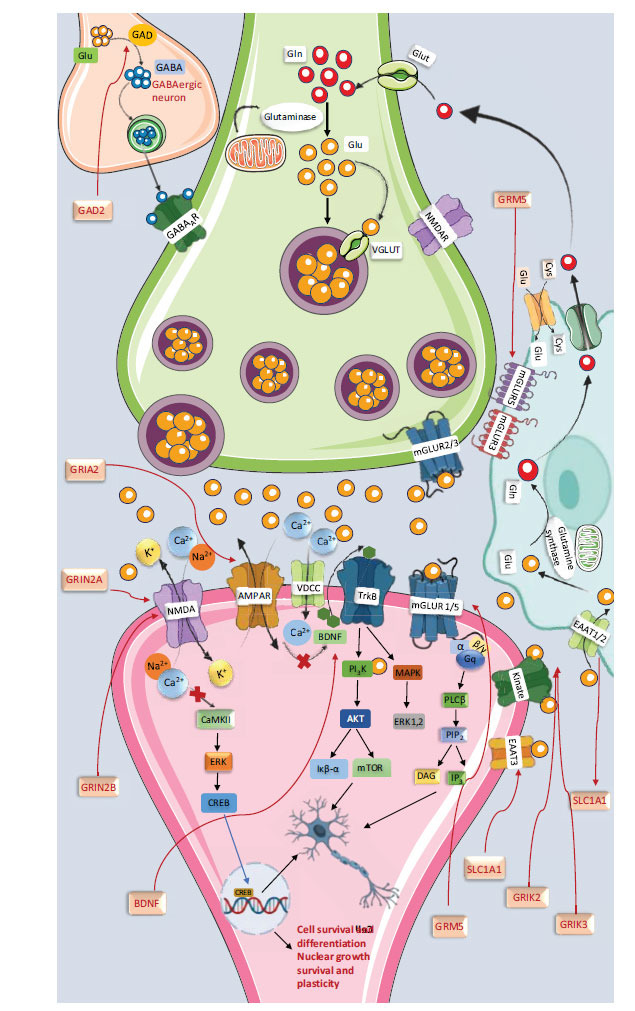
Genetic mutations in glutamatergic pathways and OCD pathogenesis. In this diagram, various gene mutations associated with glutamatergic pathways, which are responsible for the pathogenesis of OCD, are mentioned. Mutations in GAD2 genes cause the reduction of GAD, which is responsible for the production of GABA from glutamate. Mutations in the GRIA2 gene cause disturbances in the AMPAR receptor, leading to the influx of Na+ ions into the neuron. This influx of Na+ causes depolarization of the membrane, subsequently activating the NMDA receptor, which leads to an influx of Ca++ ions. Additionally, the NMDA receptor is also activated *via* mutated GRIN2A and GRIN2B genes, contributing to the progression of OCD. Due to an increased glutamate level in the synaptic cleft, the VDCC channel is activated, leading to an increased influx of Ca++ inside the neurons. This increased Ca++ *via* NMDA and VDCC receptors binds with CaMK2, affecting the level of CREB in the nucleus. Mutations in the BDNF gene cause the unbinding of BDNF with the TrkB receptor, preventing the activation of the PI3K and AKT signaling pathways, which play an essential role in cell survival and nuclear growth. The mutation in the GRM5 gene interferes with mGLUR 1/5, further leading to decreased levels of IP3-DAG, which also contributes to the pathogenesis of OCD. Mutations in the SLC1A1 gene block the EAAT3 receptor, responsible for the transfer of glutamate. While this transporter is also present in glial cells for glutamate transport, mutations prevent this process, contributing to OCD progression. Mutations in the GRIK2 and GRIK3 genes block the kainate receptor responsible for ion movement. Moreover, due to the blockade of EAAT1/2 receptors, the reuptake of glutamate into glial cells is hindered, preventing the conversion of glutamate to glutamine. Consequently, the level of glutamate increases in the synaptic cleft, furthering the progression of OCD. **Abbrevations:** GAD: Glutamic acid decarboxylase ; Glu: Glutamate; GABA: Gamma-aminobutyric acid; Gln: Glutamine; Glut: Glutamate transporter; VGLUT: Vesicular glutamate transporter; NMDAR: N-methyl-D-aspartate receptor; GRM5: Metabotropic glutamate receptor 5; Cys: Cysteine; mGLUR: Metabotropic glutamate receptor; GRIA: Glutamate ionotropic receptor AMPA subunit; GRIN2A: Glutamate ionotropic receptor NMDAR subunit 2A; AMPAR: Alpha-amino-3-hydroxy-5-methyl-4-isoxazolepropionic acid receptor; VDCC: Voltage-dependent calcium channel; TrkB: Brain-derived neurotrophic factor receptor; BDNF: Brain-derived neurotrophic factor; PI3K: Phosphatidylinositol 3-kinase; MAPK: Mitogen-activated protein kinase; CaMK2: Ca2+/calmodulin-dependent protein kinase 2; ERK: Extracellular signal-regulated kinase; CREB: cAMP response element-binding protein; AKT: Protein kinase B; Iκβ-α: I kappa B alpha; mTOR: Mammalian target of rapamycin; PLCβ: Phospholipase C beta; IP3: Inositol trisphosphate; DAG: Diacylglycerol; PUP2: Plasma membrane proteolipid transporter 2; EAAT3: Excitatory amino acid transporter 3; GRIK3: Glutamate ionotropic receptor kainate subunit 3. Symbols: 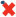 inhibition.

Many genes are implicated in neurotransmitter synthesis, storage, release, and metabolism, including serotonin, glutamate, and dopamine. Genes and variants, including TPH2 (rs4565946, rs4570625) and SLC6A4 (rs16965628, rs1315158539), are associated with synthesis, storage, and serotonin release, respectively. SLC1A1 (EAAT3) (rs301430, rs301434, rs301435, rs301979, rs3087879, rs3780412, rs7858819) and GRIN2A, GRIN2B (rs1805476) are genes that are implicated in the synthesis and transportation of glutamate, respectively [43]. OCD is associated with genes, such as SLC6A3, a dopamine transporter gene (Fig. **5**).

## 17 OTHER GENES ASSOCIATED WITH OCD

Genes distinct from those associated with neurotransmitters are accountable for the development of OCD. Oligodendrocyte lineage transcription factor 2 (OLIG2) is the receptor that controls the development and regulation of oligodendrocytes. The oligodendrocytes myelinate the central nervous system axon. OCD is characterised by OLIG2 gene overexpression in the brain's thalamus, amygdala, and caudate nuclei [180, 181]. An additional multitude of variants are correlated with OCD. This gene also contains three SNPs (rs1059004, rs762178, and rs9653711) linked to OCD in early-onset OCD trios of family-based association studies [182]. Numerous immune-related genetic variants are also implicated in *Streptococcus* or PANDA-associated paediatric autoimmune neuropsychiatric disorders.

Myelin Oligodendrocyte Glycoprotein (MOG) is an essential element of the complement cascade. It is associated with four polymorphisms, including a dinucleotide CA repeat (MOG2) and intronic single nucleotide polymorphisms C1334T and C10991T2. These polymorphisms have been identified in probands from 160 nuclear families. Tumour Necrosis Factor-alpha (TNFα), a pro-inflammatory cytokine, is implicated in various autoimmune diseases and rheumatic fever. Significant associations with both −308 G/A and −238 G/A polymorphisms were identified in a study conducted in Brazil. Susceptibility to OCD may be associated with genetic variants in MOG and TNFA, according to the results [181] (Table **1**).

## 18 RELATIONSHIP BETWEEN BRAIN CIRCUITRY AND GENETIC VARIANTS

A multitude of neuro-immuno-impairing investigations have demonstrated that the CSTC mediates OCD [181-215]. The CSTC circuit regulates movement initiation, selection, reinforcement, and reward [216]. CSTC circuit plays a critical role in brain function, encompassing key structures, like the Prefrontal Cortex (PFC), striatum (caudate nucleus and putamen), globus pallidus (internal and external segments), thalamus, and associated pathways. The PFC regulates this circuit's decision-making, planning, and emotion control., particularly the dorsolateral and ventromedial regions.

The striatum and globus pallidus modulate motor and cognitive functions through pathways, with the thalamus acting as a relay station. Dysregulation of the CSTC circuit, characterized by aberrant neurotransmitter signaling and disrupted neural connectivity, is linked to neuropsychiatric disorders, like OCD, leading to repetitive behaviors, compulsions, and difficulties in decision-making and emotion regulation.

Glutamatergic signals originating from the orbitofrontal cortex and anterior cingulate cortex stimulate the striatum in a typical CSTC circuit. These excitatory signals originating from the striatum amplify the inhibitory GABA signals transmitted to the GPi and SNr. Such a reduction in GABA release from the SNr and GPi to the thalamus induces excitatory signals to travel from the thalamus to the prefrontal cortex, thereby inducing excitatory activity.

On the contrary, the indirect pathway comprises the following components: the Subthalamic Nucleus (STN), GPi/SNr, thalamus, Globus Pallidus external segment (GPe), and ultimately the cortex. The striatum inhibits GPe, which reduces the inhibition of subthalamic nuclei. This, in turn, stimulates the activation of GPi and SNr, which subsequently inhibit the thalamus and result in a decline in cortical activation. This has a primarily inhibitory effect. OCD is characterised by an imbalance between the direct and indirect pathways, primarily overexciting the direct pathway [56]. Five subcircuits have been identified as being associated with OCD, with three primary frontal-subcortical circuits originating in the prefrontal cortex being of particular interest due to their behavioural significance. The dorsolateral prefrontal circuit, which is accountable for regulating “executive” functions, and the orbitofrontal circuit, which is involved in motivational mechanisms, are examples of such circuits. The medial component of the orbitofrontal circuit facilitates appropriate behavioural responses by integrating visceral-amygdalar functions with the organism's internal state. In contrast, the lateral component integrates limbic and emotional information [217]. The Cortico-striato-thalamo-cortical (CSTC) circuit involves multiple brain regions regulated by various neurotransmitters that control diverse functions. Polymorphisms in receptors and transporters of these neurotransmitters alter their levels in different brain areas, contributing to behaviors associated with OCD. These variations disrupt neurotransmitter signalling pathways critical for motor, cognitive, and emotional processes within the CSTC circuit, thereby influencing the development and manifestation of OCD symptoms.

## 19 GENES AND VARIANTS ASSOCIATED WITH THE PATHWAYS

As previously stated, the pathophysiology of OCD is characterised by the regulation of numerous circuits by a variety of neurotransmitters, including serotonin, dopamine, and glutamate. As a result, the development of OCD is influenced by the association of multiple genes and their variants with neurotransmitter receptors or transporters. Furthermore, the correlation between genes and brain circuits is verified by specific animal models. SAPAP3, highly expressed in the striatum and localized in the postsynaptic density at excitatory synapses, is essential for normal behavior. Deleting Sapap3 in rodents induces compulsive self-grooming and anxiety, resulting in facial hair loss and skin lesions. This deletion impairs glutamatergic transmission at cortico-striatal synapses, but reintroducing SAPAP3 *via* virus-mediated techniques restores synaptic and behavioural abnormalities, highlighting the critical role of cortico-striatal circuitry in compulsive grooming behavior [42]. Numerous association studies have been undertaken, yet none have documented a correlation between SAPAP3 variants and OCD. However, this indicates these gene variants' potential relevance to hygiene behaviour [218].

Prominently localised in the central nervous system, SLITRK proteins impact neurite outgrowth and neuronal survival. Likewise, SLITRK1 is abundantly expressed in CSTC circuitry. The anxiety levels of SLITRK1-deficient mice have been reported to be marginally elevated, displaying few behavioural abnormalities. Recently, it was also reported that rodents carrying a SLITRK5 null/reporter allele, in which the coding region was substituted with LacZ, exhibited compulsive self-grooming and increased anxiety [214].

The relationship between regional brain volumes and 519 SNPs from nine glutamatergic candidate genes, DLGAP1, DLGAP2, DLGAP3, GRIN2B, SLC1A1, GRIK2, GRIK3, SLITRK1, and SLITRK5, was investigated in a study. Two DLGAP2 single nucleotide polymorphisms (rs6558484 and rs7014992) exhibited robust associations with OFC white matter volume and other noteworthy observations in the ACC, OFC, and thalamus [219-231].

A study genotyped serotonin genes (HTR2A, HTR1B, HTR2C, SLC6A4) in 200 pediatric subjects and used structural MRI to assess brain volume in the CSTC circuit. Significant interactions were found between two SNPs in HTR2C, rs12860460 (*P* = 9.70e-8) and rs12854485 (*P* = 2.07e-6). OCD patients showed increased ACC volume, while controls showed decreased ACC volume [143, 219, 220]. Mutations in the HOXB8 gene are crucial for brain development, increased grooming behavior in mice, and Dlx5/6 gene mutations, which are important for GABA system neuron function and reduced neuronal plasticity. Various genes and their variants are associated with pathway alterations that can induce OCD [84, 221, 222]. Furthermore, there are several genes and their variants that are associated with pathway alterations that have the potential to induce OCD.

Obsessive-Compulsive Disorder (OCD) is characterized by intrusive thoughts and repetitive behaviors, linked to abnormalities in the Cortico-striato-thalamo-cortical (CSTC) pathway. Neuroimaging studies have revealed structural and functional changes in OCD patients. CT and MRI show decreased grey matter in the caudate nucleus and increased grey matter in the Orbitofrontal Cortex (OFC) [223]. PET and SPECT studies report hyperactivity in the OFC, caudate nucleus, Anterior Cingulate Cortex (ACC), and thalamus [224]. fMRI indicates decreased activation in CSTC structures and the parietal cortex. Metabolic studies, such as MRS, reveal a hyperglutamatergic state, while DTI shows altered white matter connectivity, particularly in the corpus callosum and cingulate bundle, suggesting disrupted brain communication [5, 225]. Emerging techniques, like Near-infrared Spectroscopy (NIRS) and Magnetoencephalography (MEG) add more data. NIRS studies show reduced prefrontal cortical activity, while MEG reveals phase-specific activity changes in the Dorsolateral Prefrontal Cortex (DLPFC), insula, and other regions. These findings enhance our understanding of the neural mechanisms underlying OCD and have implications for targeted treatment approaches [226, 227].

Neuroimaging studies involving OCD patients have revealed structural and functional abnormalities in the CSTC circuit, such as decreased caudate nucleus volume and hyperactivity in the OFC, indicating a dysfunctional loop and guiding targeted therapies, like medications and neurostimulation. Emerging methods, like NIRS and MEG, can help target treatments, like deep brain stimulation, promising relief for treatment-resistant OCD cases [228, 229].

OCD may result from disrupted brain communication, particularly in the prefrontal cortex-limbic system circuit, leading to emotional dysregulation and compulsions. Imbalances in dopamine and glutamate contribute to intrusive thoughts and difficulty in learning new responses. Genetic, epigenetic, and potentially inflammatory factors influence brain development and function, impacting these circuits and neurotransmitters. The neurodevelopmental theory suggests that disruptions in brain development in utero or early life lead to neuropsychiatric symptoms later [230, 231].

OCD is a neurological condition influenced by genetic and environmental factors. Genetic variations in serotonin, glutamate, and dopamine pathways contribute to neurotransmitter dysregulation, disrupting cortico-striato-thalamo-cortical circuits. Environmental factors, like stress and inflammation, disrupt CSTC circuitry, heightening OCD vulnerability. Decreased antioxidants cause increased oxidative stress and neuron damage. The gut-brain axis pathway is implicated, with decreased beneficial bacteria and increased pathological strains damaging the gut epithelial barrier [231, 232].

## 20 GENE-ENVIRONMENT INTERPLAY

### 20.1 Role of Environmental Factors in Triggering OCD

Apart from genetic predisposition, multiple environmental factors contribute to OCD development, including stress, trauma, co-morbid conditions, socioeconomic status, streptococcal infections (PANDAS/PANS) [221, 233], parental age, and birth complications. A study examining OCD patients revealed links among alexithymia, developmental trauma, attachment, and OCD severity [234, 235]. Positive correlations were found between childhood trauma and attachment avoidance, leading to increased alexithymia, which was associated with greater OCD severity and symptom quantity. In a study on 160 OCD patients, 55.6% had a history of childhood trauma, while 44.4% did not. The Beck Depression Inventory, Childhood Trauma Questionnaire, and Beck Anxiety Inventory were used to examine these correlations. The findings suggested that the association between childhood trauma and OCD should be reevaluated, considering additional factors, like comorbid conditions [235].

Research comparing 68 OCD patients to 70 controls revealed a significant association between OCD and pregnancy-related risks (*p* ≤ 0.001). Another study found correlations between childhood maltreatment (emotional and sexual abuse) and OCD among 68 patients. In a study assessing childhood trauma's impact on OCD in females (n=74) *versus* controls (n=31), trauma, particularly emotional, physical, and sexual abuse, was linked to OCD onset [194].

A study using the Swedish Medical Birth Register examined perinatal factors and found an inverse dose-response relationship among gestational age, birth weight, and OCD risk. Higher frequencies of perinatal events increased OCD risk. Lower maternal age was associated with higher OCD risk in males, while socioeconomic status was more influential in females [222, 236, 237].

A study on 442 women from the US and the Netherlands examined the relationship between reproductive events and OCD symptoms. It found OCD onset within a year after menarche in 13%, during pregnancy in 5%, postpartum in 4.7%, and at menopause in 3.7%. Exacerbation of preexisting OCD occurred in 32.7% during menopause, 37% during pregnancy, and 46.6% postpartum. Conditions, like PANDAS, PANS, autoimmune encephalitis, systemic autoimmune disease, and autoimmune CNS disorders, often associated with infections, such as streptococcal, Influenza, Epstein-Barr virus, and Borrelia burgdorferi, were linked to increased OCD risk, highlighting the role of environmental factors in OCD development [160, 232, 238, 239].

### 20.2 Gene-environment Interactions in the Context of Polygenic Traits

Gene-environment interaction (G × E) pertains to neuropsychiatric disorders, including OCD, where environmental factors influence genetic effects to cause variations in genetic expression. Existing research has demonstrated that the susceptibility to OCD is increased through the interaction of environmental factors (specifically, childhood trauma and stressful life events) and gene polymorphisms (including BDNF, MAO, COMT, 5HTT, PGRN, SMAD4, and SLC1A1) [232].

A study examined the interaction between childhood trauma and the PGRN gene, a key regulator of brain inflammation, in 484 OCD patients and 368 healthy controls. Genotyping four single nucleotide polymorphisms revealed that the TCA haplotype block (rs3859268, rs3785817, rs2879096) correlated with an increased likelihood of developing OCD. Severe childhood trauma was linked to more pronounced OCD symptoms, indicating that the PGRN-trauma interaction may contribute significantly to OCD and depressive symptoms in patients [232].

Another study investigated the BDNF Val66Met variant in OCD patients, finding that while the variant itself was not directly correlated with OCD, the Met-allele increased OCD risk when interacting with childhood emotional maltreatment [160]. Additionally, a meta-analysis focused on the TNF-α gene (chromosome 6) involving 1073 controls and 435 cases found a significant correlation between the TNF-α-238G/A polymorphism and a reduced likelihood of developing OCD, suggesting a potential protective function (OR = 0.22, 95% CI = 0.06-0.73, *P* = 0.014; OR = 0.21, 95% CI = 0.06-0.71, *P* = 0.012) [147, 239]. A study on 238 OCD patients investigated the interaction between environmental stress and genetic factors, specifically SLC1A1 variants, with respect to pharmacological resistance in OCD. The single-marker association study identified a significant association involving the SLC1A1 gene SNP (rs3087879), suggesting that genetics and environment jointly influence pharmacological resistance in OCD [240]. A pilot study examined gene-environment interactions involving MAOA, MAOB, and COMT genes with OCD and childhood trauma. Ten polymorphisms (three COMT variants, three MAOA variants, and four MAOB variants) were genotyped in OCD patients and controls. The results revealed a significant correlation between sexual abuse, a form of childhood trauma, and all three genes through haplotype-environment interaction analyses [157].

## 21 IMPLICATIONS FOR DIAGNOSIS AND TREATMENT

### 21.1 Potential for Genetic Markers in Early Diagnosis

Numerous studies have already identified specific genetic markers that can be utilised for the early detection of OCD. Furthermore, numerous other genetic markers remain inadequately understood, but they may also function as genetic markers for OCD. Modern methodologies, including Genome-wide Association Studies (GWAS), examine the genomes of numerous individuals to identify genetic markers; for instance, four studies have identified the genes NRXN1, HTR2A, CTTNBP2, and REEP3 as being significantly associated with OCD [210]. In addition, the PRS is utilised to estimate the individual genetic risk for a particular trait or disease following the GWAS analysis [180].

A significant association between symptoms of OCD and a substantial genetic marker located in the BLOC-1-related complex subunit 8 (BORCS8 or MEF2BNB) genes was established in a Genome-wide Association Study (GWAS) encompassing 6931 individuals from the Netherlands Twin Registry [237, 240, 241]. An additional investigation centered on children and adolescents (n = 5018) discovered a noteworthy genetic locus within the PTPRD gene [241]. Several studies have indicated a potential correlation between OCD and chromosome 9p24. Furthermore, numerous genes, including SLC1A1, DLGAP1, COMT, and MAO, and certain other variants that are linked to OCD may also serve as genetic diagnostic markers for the disorder.

### 21.2 Various Clinical Tests for OCD

Ongoing research focuses on genetic tools and assays for detecting genes associated with neuropsychiatric disorders, including OCD, ADHD, and schizophrenia. The National Library of Medicine's Genetic Testing Registry lists 23 genetic diagnostic tests, including 13 deletion/duplication tests, 20 sequence analyses of coding regions, 3 target variant analyses (*e.g*., Huntington disease repeat expansion analysis), and one methylation analysis (*e.g*., Prader-Willi syndrome). Limited discussion covers approximately 20 tests utilizing sequencing analysis of the entire coding region.

### 21.3 SLC6A4 - NGS Including CNV Analysis

Next-generation sequencing is a potent instrument for identifying SLC6A4 gene mutations. This technology permits precise Copy Number Variation (CNV) analysis, which can identify deletions or modifications in the SLC6A4 gene. Genetic alterations of this nature might contribute to the development of neurological and psychiatric disorders.

### 21.4 Postnatal Susceptibility to Memory Impairment Caused by the BDNF Gene (Sequence Analysis-all Coding Exons)

Sequencing the complete protein-producing segment of a gene enables sequence analysis of all coding exons to detect mutations within this critical area. Mutations in the BDNF gene can potentially increase the likelihood of postnatal memory difficulties; therefore, sequence analysis of all coding exons is necessary to identify these mutations.

### 21.5 Protection Against Obsessive-compulsive Disorder (164230; BDNF gene), Autosomal Dominant (Postnatal Sequencing Analysis-all Coding Exons)

Essential for neuronal development and mood regulation, BDNF gene mutations are associated with an increased risk of OCD. Through sequence analysis of every coding exon, gene mutations associated with an increased susceptibility to OCD can be identified.

### 21.6 Autosomal Dominant Obsessive-compulsive Disorder (164230; SLC6A4 Gene), Postnatal (Sequence Analysis-all Coding Exons)

Essential for serotonin transport and mood regulation, SLC6A4 gene mutations are associated with an increased risk of OCD. By analysing the sequences of all coding exons, gene mutations associated with increased susceptibility to OCD can be identified. In addition, there are single gene-based tests, including the BDNF single gene test, SLC6A4 single gene, and HTR2A single gene, which are accountable for deletion/duplication analysis and sequence analysis of the entire coding region, respectively. As we investigated, two elite genes, namely SLC6A4 and HTR2A, which are significantly associated with OCD, were identified using 31 and 36 assays, respectively (Obsessive–compulsive disorder–NIH Genetic Testing Registry (GTR)–NCBI) (Table **2**).

### 21.7 Personalized Treatment Approaches based on Genetic Insights

Personalized medicine, or precision medicine, tailors medical interventions based on individual attributes, utilizing molecular and genetic profiles to optimize therapeutic strategies. In OCD, genetic variability affects responses to standard treatments, underscoring the need for personalized approaches. Genetic testing identifies variations linked to OCD risk, guiding medication selection, dosage, and side-effect monitoring [242]. Pharmacogenomics customizes treatments to enhance efficacy and reduce adverse effects by considering genetic variants' metabolic impacts [243].

Neuroimaging techniques, such as EEG, fMRI, PET, and SPECT, aid in personalized OCD treatments [244]. With about 50% of patients not responding to psychotropic medications, pharmacogenetics is crucial for managing psychiatric disorders, like OCD [245]. Pharmacogenetic findings inform clinicians about drug metabolism, efficacy, and side effects. For instance, SLC6A4 gene variants, particularly the 5-HTTLPR region, impact SSRI treatment responses [246-251]. Carriers of the S allele, linked to higher serotonin levels and reduced reuptake efficiency, show a diminished response to SSRIs and increased adverse effects compared to L/L or L/S genotypes [252, 253].

Numerous medications, including Tricyclic Antidepressants (TCAs) and Selective Serotonin Reuptake Inhibitors (SSRIs) used to treat OCD, are metabolized by CYP genes. Variations in these genes can affect drug metabolism rates, influencing medication efficacy and exhibiting adverse effects [38]. Polymorphisms in the CYP2D6 gene can impair enzyme functionality, impacting the metabolism of SSRIs (fluoxetine, paroxetine, fluvoxamine), venlafaxine (SNRI), and amitriptyline (TCA), with CYP2D6 allele variants producing different metabolizer phenotypes, from extensive to deficient [248].

The CYP1A2 gene, responsible for encoding the CYP1A2 enzyme, affects the metabolism of about 24% of antidepressants, including agomelatine, escitalopram, venlafaxine, duloxetine, and mirtazapine [246]. Specific Single Nucleotide Polymorphisms (SNPs) in CYP1A2, including rs4646425, rs2069521, and rs4646427, alter escitalopram metabolism, increasing the risk of adverse effects, like fatigue and nausea, during initial therapy [250].

Additionally, other enzymes, such as COMT and MAO, are influenced by genetic mutations that affect antidepressant functionality. The MAOA rs979605 polymorphism may have a sex-specific effect on antidepressant response, while the COMT gene polymorphism rs4680 (Val158Met) is associated with responses to SSRIs paroxetine and fluoxetine [251, 252].

When patients do not respond to SSRIs, personalized treatments, such as clomipramine, are prescribed, taking into account individual pharmacokinetics, pharmacodynamics, and genetic deficiencies in CYP2D6 enzyme activity, which affects drug clearance. This personalized approach optimizes the benefit-to-risk ratio, improving the likelihood of successful OCD treatment [253]. Pharmacogenetics, personalized medicine, GWAS), Polygenic Risk Scores (PRS), and neuroimaging, all contribute to tailored treatments. Personalized medicine has the potential to significantly enhance treatment outcomes and quality of life for OCD patients by leveraging genetic data and advanced technologies in order to provide more effective, patient-focused care [254].

## 22 CURRENT THERAPEUTIC APPROACHES AND ONGOING RESEARCH ON OCD

The most commonly used treatment approaches for OCD are behavioural therapies, pharmacotherapies SSRI, antidepressants, antidopaminergic and glutaminergic agents, rTMS, and deep brain stimulation. The first line of treatment starts with behavioural therapies and ends with the DBS based on resistance to other methods and the severity. Additionally, various personalised treatments and digital apps (*e.g*., mobile apps, virtual reality exposure therapy) and the above-discussed therapies are undergoing clinical trials.

## 23 BEHAVIOURAL THERAPIES

### 23.1 Cognitive Behavioural Therapy

Cognitive Behavioural Therapy (CBT) is a therapeutic method that helps individuals identify and modify maladaptive thought patterns and behaviors to alleviate psychological distress. It is widely recognized as the most effective method for treating OCD and other psychological conditions, using techniques, like relaxation techniques and cognitive restructuring [255].

As a form of CBT, Exposure and Response Prevention (ERP) is the most effective treatment for OCD. In a study involving fourteen children and adolescents diagnosed with OCD, six patients received CBT alone, while eight patients received concurrent medication (serotonin reuptake inhibitors). Using the Y-BOCS to assess OCD symptoms, it was determined that CBT reduced OCD symptoms effectively [256]. Comparing the efficacy of adding cognitive-behavioural therapy (Exposure and Ritual Prevention, EX/RP) or risperidone medication to ongoing Serotonin Reuptake Inhibitor (SRI) treatment for adults with moderate to severe OCD, a randomized clinical trial with 100 participants (NCT00389493) found that EX/RP was significantly more effective in reducing OCD symptoms than both risperidone and placebo, and patients receiving EX/RP experienced a mean reduction in Y-BOCS score. While receiving EX/RP compared to risperidone and a placebo, insight, functioning, and quality of life were all found to be improved significantly [63]. To compare the efficacy of family-based CBT and family-based relaxation therapy in young children (5-8 years old) with OCD, an additional clinical trial (NCT00055068) was undertaken. 42 OCD patients were randomized to CBT (12 sessions) or Relational Therapy (RT) groups (12 sessions). A significant proportion of adolescents who underwent CBT achieved clinical remission and a reduction in OCD symptoms [257].

An additional clinical trial, designated NCT00000386, aimed to compare the efficacy of structured family intervention (FCBT) and exposure-based cognitive-behavioural therapy with that of relaxation training and psychoeducation. The objective of this trial was to assess the effects of the intervention on juveniles with OCD in terms of functional impairment improvement, symptom severity reduction, and family accommodation of symptoms. FCBT demonstrated notably superior response rates to Pain Reprocessing Therapy (PRT), as determined by Intent-to-treat (ITT) and completer analyses. Parent-reported accommodation of symptoms, child-reported functional impairment, and OCD severity all improved marginally more as a result of FCBT. Additionally, FCBT reduced OCD symptoms and attained a higher clinical remission rate (42.5%) than PRT (17.6%) [258]. To compare the efficacy of Internet-based Cognitive Behaviour Therapy (ICBT) with therapist support for treating OCD to that of an attention control condition, one more clinical trial (NCT01525576) was undertaken. A total of 101 OCD patients were assigned at random to receive either ten weeks of ICBT or the control condition. Although both interventions ameliorated symptoms of OCD, ICBT produced notably more substantial improvements in comparison to the control condition [259]. Comparing Motivational Interviewing (MI) in conjunction with exposure and response prevention (EX/RP) to EX/RP alone, another clinical trial (NCT00316316) involving 30 individuals of varying ages discovered a significant reduction in the severity of symptoms as measured by Y-BOCS. In addition, the BAI, BDI, and Global Assessment of Functioning (GAF) all demonstrated substantial improvements for both groups. This study suggested that MI may not provide additional therapeutic benefits for OCD beyond EX/RP alone [260]. An investigation was conducted on the feasibility and preliminary efficacy of Attention Training (AT) as a treatment for childhood OCD through a clinical trial (NCT01708226). The results of BAI and Children's Depression Inventory (CDI) revealed a statistically significant reduction in Y-BOCS.

To alleviate the symptoms of OCD, numerous clinical trials are currently underway; the clinical trial identifiers (NCT05467683, NCT03855943, NCT05284435, NCT 05528224, NCT04527302, and NCT04071990) are listed in Table **3**.

### 23.2 Brain Stimulation Therapy in OCD

Traditional OCD therapy includes CBT and psychotropic medications, but 10% of patients still experience severe symptoms. Neurosurgical interventions, like Deep Brain Stimulation (DBS), are being explored as potential alternatives to ablative surgery, targeting dysfunctional brain regions [261].

The progression of these methodologies indicates a transition towards DBS as a prospective therapeutic approach for refractory OCD. Nuttin *et al.* reported the initial use of DBS to treat OCD in patients resistant to conventional treatments in 1999 [262]. DBS is a neurosurgical technique characterised by the stereotactic implantation of electrodes designed to transmit electrical impulses to specific subcortex or deep cortex regions. An extension cable links a pulse generator surgically implanted in the thorax to an electrode affixed to the brain through the skin of the scalp and neck [263]. DBS primarily stimulates the Ventral Striatum (VS), the Anterior Limb of the Internal Capsule (ALIC), the Bed nucleus of the Striata Terminalis (BST), the Nucleus Accumbens (NAc), and the Medial Forebrain Bundle (MFB) [264] in the striatal region [265].

As shown in the table, numerous clinical trials have been conducted on brain stimulation for OCD, while others are still in the planning stages. In a clinical trial (NCT 01135745) involving 32 participants, DBS was utilised to reduce the Y-BOCS score [266]. A further clinical trial (NCT01807403) was undertaken to compare the efficacy of stimulating distinct brain regions, including the subthalamic nucleus and caudate nucleus, in patients diagnosed with severe and resistant OCD. The results indicated that stimulating the Subthalamic Nucleus (STN) resulted in a significantly more significant reduction in Y-BOCS (46.5% *vs*. 32.4%), suggesting STN stimulation to be a more effective treatment option than stimulating the caudate nucleus (NCT01807403) [11]. The results of an additional clinical trial (NCT00169377) involving stimulation of the subthalamic nuclei revealed a substantial improvement in Y-BOCS. Two additional clinical trials (NCT01540305, NCT00822601) utilising repetitive Transcranial Magnetic Stimulation (rTMS) determined that it reduced OCD symptoms by a significant margin. A decrease in the Y-BOCS score indicates an OCD symptom reduction. In addition, numerous ongoing clinical trials (NCT03211221, NCT05331937, NCT04217408, NCT03211221) are investigating the potential therapeutic benefits of brain stimulation for OCD (https://clinicaltrials.gov/).

### 23.3 Pharmacotherapy for OCD

Efforts to treat OCD in the 1960s and 1970s were primarily frustrated by the lack of a specific medication for the disorder; instead, existing medications intended for other conditions, such as antidepressants and antipsychotics, were utilised [274]. As the first effective treatment for OCD, clomipramine, a tricyclic antidepressant with potent Serotonin Reuptake Inhibition (SRI) properties, was developed [249]. SSRIs, like fluoxetine and fluvoxamine, revolutionized OCD treatment in the 1980s-1990s with precise serotonin reuptake inhibition and better toleration than clomipramine. Despite clomipramine's slightly better effectiveness, its adverse effects limited initial use, making it a significant alternative when SSRIs proved ineffective in OCD treatment [249]. When patients develop resistance to SSRIs or SRIs, medication augmentation with antidepressants (venlafaxine, paroxetine) or antiepileptic agents (topiramate, lamotrigine) is the preferred approach.

In addition, numerous drugs are undergoing clinical trials, including cannabinoids, dopaminergic agents, glutamate agents, serotonergic agents, probiotics, nutraceuticals, and dopamine, norepinephrine, and serotonin agents. Some of these drugs are currently in the ongoing phase of clinical trials. Eleven individuals with OCD participated in a clinical trial (NCT02911324) involving the cannabinoid agent nabilone; significant improvements in extinction learning and reductions in repetitive behaviours were observed [267-275]. Twenty individuals participated in a clinical trial of dopaminergic agents, including tolcapone (NCT03348930), which found significant improvement in OCD symptoms; sixty adults participated in another clinical trial of dopaminergic agents, including methylphenidate hydrochloride (NCT02194075), which found a significant reduction in OCD symptoms [276]. An additional clinical trial (NCT00471588) involving dopamine D1 and D2 receptor agonists and antagonists (pramipexole and amisulpride) was conducted on 52 adults and older adults. The results indicated that pramipexole significantly increased symptoms of OCD, whereas amisulpride decreased OCD symptoms as measured by the Y-BOCS score. Clinical trials involving serotonergic agents, such as the 14-adult ondansetron (NCT00796497) clinical trial study, demonstrated a reduction in OCD symptoms as measured by a decrease in the Y-BOCS score. This rating was derived from a selective 5-HT3 (serotonin) receptor antagonist [277]. An additional ondansetron trial (NCT03239210) involving 110 adults resulted in a reduction in OCD symptoms as well as enhancements in BDI, BAI, and GAF. An additional clinical trial involving 41 children on sertraline (NCT02797808) revealed a substantial reduction in the Y-BOCS scale and improved connectivity in the left putamen. Clinical trials on glutaminergic agents were also conducted; for instance, one (NCT01695291) involving 31 child adults on minocycline found significant improvement in OCD symptoms; Y-BOCS also exhibited ameliorated hoarding symptoms [277]. A clinical trial involving 23 adults on topiramate demonstrated notable enhancements in Y-BOCS, BDI, and BAI scores. These improvements imply that topiramate may be effective in reducing symptoms associated with depression and anxiety. An additional clinical trial involving a glutaminergic agent, such as pregabalin (NCT00994786), demonstrated a substantial reduction in Y-BOCS and amelioration of symptoms associated with OCD. Hoarding symptoms decreased in four individuals who were administered the norepinephrine-dopamine agent methylphenidate ER (NCT01100268), a NET and DAT transporter inhibitor, as part of a clinical trial [277]. A trial on the dopamine-serotonin-norepinephrine antagonist risperidone (NCT 00389493), a D2 and 5HT2A receptor antagonist, was conducted on 100 adults and older adults. The trial demonstrated this medication to have a superior effect on OCD [63]. A subsequent trial on N-acetyl cysteine, administered to 40 adults, revealed a significant decrease in Y-BOCS [278]. There have been previous trials conducted that have demonstrated the substantial efficacy of these medications in the treatment of OCD. In addition, there are numerous ongoing clinical trials, including phase 2 of a clinical trial for the dopaminergic agent tolcapone (NCT05624528), which aimed to alleviate symptoms of OCD. Several cannabinoids, including nabilone (NCT04880278), dronabinol (NCT01093976), and Epidiolex (NCT04978428), are also in the experimental phase. In addition, phase 1 clinical trials are currently underway for certain serotonin agents, including psilocybin (NCT05546658, NCT05370911, NCT03300947, NCT03356483), which acts on 5-HT(1A) and 5-HT(2A/2C) receptors. Additionally, two more clinical trials are ongoing for sertraline (NCT04539951) alone and sertraline+fluvoxamine (NCT04336228), both being SSRIs. A clinical trial involving escalopram (NCT04336228), an SERT, is also ongoing in an effort to reduce OCD symptoms. Some glutaminergic agents, including nitrous oxide gas (NCT03826693), which targets AMPA and NMDA receptors and is believed to alleviate symptoms of OCD, are undergoing clinical trials. Troriluzole (NCT03299166), an additional glutaminergic agent that functions as an NMDA receptor antagonist and modulates the glutamate transporter, is also in clinical development. Clinical trials are underway for riluzole (NCT00523718), an additional glutaminergic agent that functions as an NMDA receptor antagonist and inhibits voltage-gated calcium channels. Several clinical trials are also underway for ketamine (NCT01100255, NCT01172275, NCT05565352), a non-competitive inhibitor of the NMDA receptor. Dextromethorphan (NCT05565352) is also undergoing clinical trial as a nonselective SRI (https://clinicaltrials.gov/.). In addition, several anti-inflammatory and immunomodulatory compounds, including naproxen sodium (NCT04015596), a non-selective inhibitor of COX1 and COX2, are undergoing clinical trials to be utilised in PANDA [279]. In addition, clinical trials for rituximab (NCT03983031, NCT04323566) are currently being conducted in an effort to reduce OCD symptoms [280]. The COX-2 inhibitor celecoxib is also a clinical trial subject (NCT04786548 and NCT04673578) [281-288]. In addition to these ongoing clinical trials, numerous others are in progress with the potential to provide treatments for OCD.

Novel therapeutic approaches, such as Virtual Reality Exposure Therapy (VRET), use virtual reality to create safe, controlled environments for OCD triggers, offering a more immersive experience than traditional exposure therapy and potentially leading to faster improvement for some patients. Acceptance and Commitment Therapy (ACT) helps individuals accept their thoughts and feelings without judgment and commit to a meaningful life, which can be beneficial for managing anxiety associated with OCD [289]. Other novel techniques include glutamate-focused medications and targeting specific proteins. These pharmacological approaches aim to address aspects of brain chemistry involved in OCD and are still under study. Deep Transcranial Magnetic Stimulation (dTMS), ketamine, and Theta Burst Stimulation (TBS), the advanced neuromodulation techniques, use magnetic fields or low-dose medication to stimulate brain regions linked to OCD [290]. These novel techniques are in various research stages. Some of these techniques are also mentioned in Tables **3** and **4**, but are not widely available yet.

Cognitive Behavioural Therapy (CBT) with Exposure and Response Prevention (ERP) remains the gold standard for OCD treatment [291]. Medications, like Selective Serotonin Reuptake Inhibitors (SSRIs), can also be crucial. However, recent years have seen the rise of mobile-based technologies specifically designed to aid OCD management.

The mobile apps also offer a variety of features, including psychoeducation, self-monitoring tools, ERP exercises, relaxation techniques, and cognitive restructuring guidance. While not replacing professional treatment, these apps can provide convenient, accessible, and sometimes affordable support. Habit reversal apps and biofeedback apps offer additional options for managing compulsions and anxiety.

## 24 FUTURE DIRECTIONS IN OCD RESEARCH

### 24.1 Advancements in Genetic Research Methodologies

Genetics is the study of heredity and DNA. Significant advancements occurred during the 20^th^ century. Later, technological advancements and the Human Genome Project propelled a molecular comprehension that substantially increased our knowledge of genetic factors and their influence on human life and diseases. Genetic data have been collected with the help of twin and family studies [288, 292-303]. There are first and second-line treatments for the patients of OCD, but the symptoms are still impaired. So, another SRI needs to be discovered to relieve the symptoms of OCD. The Randomized Controlled Trial (RCT) and meta-analytic evidence are organised when the partial response is achieved after first-line treatment. The RCT is performed to support the synergistic effect of second-generation antipsychotics [304, 305]. According to Brakoulias and Stockings, risperidone can be used to augment and relieve the symptoms. Other augmentation agents used in OCD are aripiprazole and quetiapine [51].

The deficiencies of earlier techniques, such as RFLP and SSCP, prompted the development of DNA sequencing. Sanger sequencing supplanted Maxam-Gilbert sequencing, which was renowned for its efficiency. Subsequently, automation propelled the sequencing procedure to new heights, facilitating the expeditious and precise sequencing of the human genome [286].

Despite challenges, like invoicing, consent, and data interpretation, NGS technology is improving disease diagnosis and noninvasive prenatal testing. The progression from karyotyping to array-CGH exemplifies the technological revolution in genetic diagnostics, with future developments potentially leading to genome-based identification cards [287].

Significant progress in molecular biology has been made with the aid of gene editing technologies, such as restriction Transcription Activator-like Effector Nucleases (TALENs), endonuclease digestion, and the Clustered Regularly Interspaced Short Palindromic Repeats (CRISPR-Cas) system [288]. CRISPR/Cas9 genome editing tools present prospects for precise therapeutic interventions and progress in early diagnosis. CRISPR/Cas9, a molecular tool for precise gene editing, holds significant potential in OCD research. It can create OCD cell models by introducing genetic mutations associated with OCD risk into healthy cells, enabling the study of these mutations' effects on brain development and function. Additionally, CRISPR allows functional analysis of genes by editing them in neurons from OCD patients or healthy controls, helping to identify how specific genes affect neuronal communication, circuit formation, and OCD-related behaviors [303].

Advancements in OCD research include enhanced Genome-wide Association Studies (GWAS), polygenic risk scores, next-generation sequencing technologies, and gene-environment interactions. Researchers are also exploring gene therapy, an experimental approach to treating neurological disorders that involves gene deletion, silencing, and modification of defective genes. This interdisciplinary strategy aims to alleviate symptoms and prevent disease progression [305].

Raison and Miller showed the effect of inflammatory cytokines on the brain circuitry [304]. Nicholson and his colleagues established the link between the pathogenesis of OCD and immunological dysfunction. The OCD patients showed increased levels of basal ganglia antibodies [248, 260, 300-311]. So, immunomodulatory therapy is the newer therapeutic approach for OCD. Antimicrobial agents, nonspecific nonsteroidal anti-inflammatory drugs, and immunological modulators, like celecoxib, have provided some positive results too [312, 313]. Cognitive therapies and rational emotive therapy are also tried for OCD. Recently, wave therapies have also been used in several psychiatric conditions, like OCD. According to Rupp and colleagues, cognitive restructuring and mindfulness show improvements in the BOCS score compared to waiting list controls. SSRIs or CBT have no effect on about one-quarter of OCD patients, so the advancements in pharmacogenetics can be a positive approach for the patients [308, 314]. Various pharmacogenetic approaches have been conducted using GWAS on the association between candidate genes and drug response in patients with OCD. Different candidate genes have been discovered with the help of advanced pharmacogenetics methods. Advancements in DBS techniques can provide deeper knowledge about the optimal target in DBS. The advancement has been made in this area till now as the target has been changed to a white matter bundle that connects to several regions of the symptomatic network, which was earlier thought to be grey matter. More studies are focused on getting more profound knowledge about the precise location and importance of BNST [315-320]. To find new biomarkers that can aid in programming and closed-loop stimulation, the NIH Brain Research has funded the two projects through Advancing Innovative Neurotechnologies (BRAIN) (NCT03457675, NCT03184454) [311].

As the treatment has been improved lately, many areas need to be improved. The main issue is treatment dissemination, particularly for CBT and EX/RP [312]. Another issue is limited augmentation treatment options. Few newer approaches have been introduced, which have provided better responses. The more recent approaches include targeting the extinction learning core to EX/RP with D-cycloserine, a partial agonist at the NMDA receptor in the amygdala and other areas, which have provided better results in both young individuals and adults. Lastly, efforts need to be made for the research on the impact of OCD treatment on comorbid conditions [315]. With the improvement in newer technologies and therapeutic strategies, the symptoms of OCD can be reduced, which can ultimately help researchers to decrease the progression of the disease (Table **5**).

### 24.2 Integration of Genetics with Other Research Avenues

The integration of genetics with other research domains pertains to amalgamating genetic information with clinical and environmental data, among others, to acquire a more holistic comprehension of diverse phenomena. This methodology has been implemented to comprehend further a multitude of scientific domains, such as neuropsychiatric research and biodiversity. The analysis of neuropsychiatric disorders is aided by integrating genetics data, including candidate gene association, genome-wide association, polygenic risk score, copy number variant, and exome sequencing information.

## CONCLUSION

This review has explored the pathological mechanisms, therapeutic approaches, and future directions of OCD, a disorder influenced by genetic, environmental, and neurotransmitter factors. The CSTC circuit, a key pathway, is a key component of OCD, involving neurotransmitters, like serotonin, dopamine, glutamate, and GABA. Genetic mutations can disrupt the CSTC circuit, causing the disease to progress. Environmental factors, like stress and trauma, exacerbate the condition. Over fifty genes and their variants have been linked to OCD, with HTR2A and SLC6A4 being significant. Currently, therapeutic prospects for early detection are limited, but over fifteen compounds are undergoing clinical trials. Future research could focus on personalized medicine and brain stimulation techniques. The review has aimed to provide an overview of recent therapeutic approaches and gene variants, contributing to the scientific community's understanding of novel compounds and therapeutic strategies.

## Figures and Tables

**Table 1:** Involvement of genetic variants in the progression of OCD.

**Sr. No.**	**Type of Genes**	**Genes/ Chromosome Location**	**Brain Region Involved**	**Genetic Variant Id and Name**	**Genetic Polymorphism**	**Normal Mechanism/ Pathway**	**Function Regulations**	**Type of Variants**	**Genetic Dysfunction in OCD/ Pathological Changes**	**References**
1	Serotonergic receptor genes	HTR1A 5q12.3	Limbic brain areas Hypothalamus and cortical areas	rs6295 NM_000524.3(HTR1A):c.-1019G>G	C/G (G dominant C>A	Serotonin signalling Gi subtype of G-protein-coupled receptors Inhibit adenylase cyclase Regulate Ca2+ release	Regulation of 5-hydroxytryptamine release Regulation of dopamine and 5-hydroxytryptamine metabolism Affected neural activity Mood and behaviour Pain perception	2KB upstream variant	G allele associated with ↑ HTR1A gene expression ↑ Serotonin 1A receptor level Alteration of emotion regulation and cognitive control	[183]
				rs10042486 NC_000005.9:g.63261329C>T	C>T			Intron variant	CT genotype significantly associated with ↑ Risk of OCD Associated with the age of onset of OCD	[135]
2		HTR1B 6q14.1	Basal ganglia Striatum Hippocampus Vascular smooth muscles	rs6296 NM_000863.3:c.861G>C	G>C	Serotonin signalling Gi subtype of G-protein-coupled receptors Inhibit adenylate cyclase	Regulate release of 5-hydroxytryptamine, dopamine, and acetylcholine Pain perception Mood and behaviour Thermoregulation Respiration Appetite control Aggression	Synonymous variant	C allele ↑HTR1B gene expression ↑ HTRIB activity ↑ Risk of OCD	[184-186]
				rs2000292 NC_000006.12:g.77457228G>A	G/A			Intron variant	Variation associated with early onset OCD	[8, 184]
3		HTR2A 13q14.2	Neocortex Caudate nucleus Nucleus accumbens Hippocampus Vascular and non-vascular smooth muscle cell	rs6311 NM_001378924.1(HTR2A):c.-329+609G>A	G/A	Serotonin signalling GPCR downstream signalling Activation of PI3K, increase ca2+ release	Aggression Perception Cognition and mood Intestinal smooth muscle contraction Arterial vasoconstriction	Synonymous Variant	An allele ↓ HTR2A gene expression Lowers serotonin level in the brain Alter emotion processing and cognitive control ↑ Compulsive behaviour ↓ Cognitive flexibility	[8]
				rs6313 NM_001165947.5:c.-78+869C>T	C>T			Synonymous Variant	↑ Compulsiveness and repetitive behaviour High prevalence of anxiety and depression comorbidity	[187, 188]
				rs2770304 NC_000013.10:g.47455365C>T	C/T			Intron variant	T allele ↑ HTR2A gene expression Higher serotonin 2a receptor level in brain ↑ Risk of OCD	[189]
4		HTR2C Xq23	Choroid plexus Cortex Limbic system Basal ganglia.	rs12860460 NM_000868.4:c.-80+52825T>C	T>C, T>G	Serotonin signalling GPCR downstream signalling Activation of PI3K, increase ca2+ release Activation of proopiomelacortin neurons Regulate the release of corticosterone	Appetite Eating behaviour Anxiogenic stimuli Stress Thermoregulation Penile erection Anxiety	Intron variant	↑ Anterior cingulated cortex volume ↑ HTR2C gene expression Serotonin 2C receptor levels in the brain: Altered serotonin signalling ↑ OCD symptoms	[143]
				rs12854485 NM_001256760.3:c.-171+48152G>A	G>A			Intron variant	↓ ACC volume in OCD	[143]
5		HTR3A 11q23.2	Amygdale Caudate Hippocampus Nucleus accumbens Basal ganglia Prefrontal cortex	rs1062613 NR_046363.2:n.177T>C	T>C	Serotonin signalling Ligand-gated ion channel fast activation, depolarising responses in neurons	Modulate the serotonin release Emotional processing and motivation Memory consolidation Motor control	Non-Coding Transcript Variant	Polymorphism ↑ risk of developing OCD	[190]
				rs1176713 NM_213621.4:c.1473A>G	A>G			Synonymous Variant	G allele responsible for ↑ risk of developing OCD ↑ Symptom severity Washing compulsions	[190]
6		HTR3B 11q23.2	Amygdale Caudate nucleus Hippocampus Thalamus	rs3758987 XM_024448767.2:c.-242-4742T>C	T/C	Serotonin signalling Ligand-gated ion channel fast activation, depolarising responses in neurons	Motor control Habit formation Decision making Spatial memory Sensory processing	Intron variant	Alter serotonin levels in the brain C allele protective action ↓ Risk of developing OCD	[190]
				rs1176744 NM_006028.5:c.386A>C	A>C			Missense variant	C allele is protective against OCD ↓ Risk of developing OCD	[190]
				rs3782025	G>A, C, T			Intron variant	Associated with ↑ harm avoidance in OCD	[190]
7		HTR3C 3q27.1	Ventral tegmental area Nucleus accumbens Central amygdale Dorsal route ganglia Myenteric plexus	rs6766410	C>A, C>T	Serotonin signalling Ligand-gated ion channel fast activation, depolarising responses in neurons	Perception of pain Facial pain perception	Missense variant	Contamination-based disgust in OCD Cleaning dimension in the female subject	[190]
8		HTR3D 3q27.1	Basal ganglia Prefrontal cortex Hippocampus Amygdale Raphe nuclei	rs6443930	G>A, G>C, G>T	Serotonin signalling Ligand-gated ion channel fast activation, depolarising responses in neurons	Serotonin synthesis Motor control Emotion and memory	Missense variant	↑ symmetry and orderliness compulsions Repetitive checking behaviours in OCD	[190]
				rs1000952	G>A, G>C, G>T			Missense variant	C allele associated with ↑ anxiety Also associated with early onset OCD	[185]
9		HTR3E 3q27.1	Myenteric plexus Dorsal route ganglia Trigeminal ganglion	rs7627615 NM_198314.2:c.256G>A NM_182589.2:c.256G>C	G>A, G>C	Serotonin signalling Ligand-gated ion channel fast activation, depolarising responses in neurons	Pain signaling Facial pain	Coding sequence variant	Related to the washing dimension and visual organization	[190]
10	Serotonin transporter	SLC6A4 17q11.2	Hypothalamus Hippocampus Thalamus Cerebellum Amygdale Medulla oblongata	rs16965628 NM_001045.6:c.-220-5492C>G	G>C	Mediates sodium- and chloride-dependent transport of dopamine, norepinephrine	Transportation of serotonin Terminating the action of 5-HT in the synaptic cleft Serotonin homeostasis in CNS Organization of cortical neurons Regulate blood serotonin level Modulate mucosal serotonin level Mood disorder Learning, memory, and attention	Intron variant	G allele associated with the development of OCD	[191]
				rs28914832 NM_001045.6(SLC6A4):c.1273A>G (p.Ile425Val)	T>C / T>G			Missense variant	An allele found to be associated with anxiety	[192]
11	Dopamine receptors	DRD1 5q35.2	Substantia nigra Basal ganglia Nucleus accumbens Olfactory bulb Prefrontal cortex Hippocampus Thalamus	rs4532 NC_000005.9:g.174870150C>T NC_000005.9:g.174870150C>G	C>G/C>T	Gs subtype of G protein-coupled receptor Stimulate adenylyl cyclase Activate cAMP-dependent protein kinases	Neuronal growth and development Motivation and reward Decision making Motor coordination Olfactory processing	Located on the promoter region of the DRD1 gene	G allele responsible for ↑ risk of developing OCD ↓ Response to SSRI T allele associated with ↑ symptoms severity	[193]
12		DRD2 11q23.2	Striatum Substantia nigra pars compacta Prefrontal cortex Pituitary gland	rs1800497 NM_178510.2(ANKK1):c.2137G>A (p.Glu713Lys)	G>A	Gi subtype of G-protein-coupled receptors Inhibit adenylate cyclase	Modulation of locomotion Reward Reinforcement and memory Learning	Missense variant	↑ Symmetric obsession Increase in symmetry-related behaviour	[194]
13		DRD3 3q13.31	Nucleus accumbens Amygdale Hippocampus Ventral tegmental area Prefrontal cortex Island of Calleja	rs6280 NM_000796.6(DRD3):c.25G>A (p.Gly9Ser)	G>A	Gi subtype of G-protein-coupled receptors Inhibit adenylate cyclase	Promote cell proliferation Emotion, motivation, and memory Dopamine release Reward processing Planning Decision making Modulation of locomotion	Missense variant	↑ Risk of developing OCD An allele associated with checking compulsion	[161]
14		DRD4 11p15.5	Mesolymbic area Frontal cortex Mid brain Amygdale	NA	48bp VNTR	Gi subtype of G-protein-coupled receptors Inhibit adenylate cyclase	Emotion and complex behaviour Cognition and emotion Memory Modulate circadian rhythms	NA	Having protective effect against OCD symptoms	[158]
15	Dopamine transportes	DAD3 3q13.31	Nucleus accumbens Olfactory tubercle Island of Calleja	rs3773679 NM_000796.6:c.384-2931G>T	C>A / C>G / C>T	Gi subtype of G-protein-coupled receptors Inhibit adenylate cyclase	Cognitive Emotional and Endocrine functions	Intron variant	↑ hoarding behaviour ↑ impulsivity and risk-taking behaviour	[162]
16				rs6280 NC_000003.11:g.113890815C>T	C>T		Cognitive Emotional and Endocrine functions	Missense variant	↑ in hoarding symptom ↑ in impulsivity and risk-taking behaviour	[195]
17		SLC6A3 5p15.33	Striatum Substantia nigra Nucleus accumbens Olfactory bulb	rs4975646 NC_000005.9:g.1433401G>A	G>A	Sodium- and chloride-dependent transport of dopamine	Influencing dopamine release Motivation and decision-making Reward process	3'-UTR polymorphisms	Influence gene expression and Serotonin transporter level Affect impulse control, mood, and cognitive function	[162]
18	Glutamate receptor genes	GRIN2B 12p13.1	Hippocampus Basal ganglia Olfactory bulb Cortical parts and Temporal parts of the brain	rs1805476 NC_000012.11:g.13714363G>C NC_000012.11:g.13714363G>T	G>C / G>T	Glutamate signaling Encodes for NMDA ion channel, Characterized by calcium permeability	Brain development Circuit formation Synaptic plasticity Cellular migration and differentiation Motor control	3 Prime UTR Variant	↓ NMDA receptors in the brain ↓ synaptic plasticity G allele ↑ risk of OCD Associated with early age onset of OCD G and T allele associated with	[196]
				rs1805501 NC_000012.11:g.13714059A>G	A>G			3 Prime UTR Variant	↓ NMDA receptors in the brain contamination and cleaning obsession A allele ↑ risk for OCD	[196]
				rs1805502 NM_000834.4:c.*1536T>C	A>G			3 Prime UTR Variant	↓ NMDA receptors in the brain contamination and cleaning obsession allele ↓ risk for OCD	[176]
				rs1805477 NM_000834.5:c.*988A>T	T>A, T>C, T>G			3 Prime UTR Variant	↓NMDA receptors in the brain contamination and cleaning obsession G allele ↑ risk for OCD	[60]
				rs1019385 XM_011520629.3:c.-683+7G>T	C>T			Intron variant	T allele associated with ordering dimensions.	[178]
				rs1806191	C>T			Intron variant	-	-
19		GRIK2 6q16.3	Dentate gyrus CA1 and CA3 pyramidal cells Cerebellum Amygdale	rs1556995 NM_021956.4:c.1524+9977C>G	C>A, C>G,	Kinate family glutamate receptor, Function as ligand-gsted ion channels Glutamate binding causes the opening of the cation channel	Regulate postsynaptic excitatory currents in CNS regions Mediate endocytosis and synaptic transmission Memory formation	Intron variant	↓ Amount of kinate receptors ↓ Synaptic plasticity GG phenotype is associate with earlier age onset of OCD	[197, 198]
20		GRIK3 1p34.3	Hippocampus Amygdale Prefrontal cortex Cerebellum	rs1002656 NC_000001.10:g.37192741C>T	C>T	Kinate family glutamate receptor Function as ligand-gated ion channels	Role in excitatory synaptic transmission	NA	Contamination and washing compulsions Symmetry obsession and ordering compulsions Hoarding behaviour	[199]
21		GRIA1 5q33.2	Hippocampus Neocortex Thalamus Cerebellum	rs2963944 NC_000001.10:g.37192741C>T	A>C, A>G, C>T	Glutamate signalling Bind to AMPA ionotropic glutamate receptor, cation channel opening,	Modulate synaptic transmission and plasticity Influencing learning Memory formation Motor control Coordination and balance	Intron variant	Impaired neural function in OCD Alter neuronal activity metabolism or myelin formation	[179]
				rs707176 NM_000827.4:c.531T>C	T>C			Synonymousvariant	Impaired neural function in OCD Alter neuronal activity metabolism or myelin formation	[179]
22	Glutamate transporter genes	SLC1A1 9p24.2	Hippocampus Cortex Basal ganglia cerebellum	rs301430	T>A / T>C / T>G	Glutamate signaling Encoded for Excitatory Amino Acid Transporters (EAATs)	Production of glutamate Protection against oxidative stress Glutamate removal and release from synaptic cleft Sodium-dependent amino acid transport Memory and language processing Motor coordination and balance	Synonymous Variant	C haplotype associated with personal distress	[135, 200]
				rs301434 XM_011518007.2:c.1263-956C>G XM_011518007.2:c.1263-956C>T	G>A C>G, C>T			Intron variant	G haplotype increases hoarding behaviour in a woman T haplotype associated with personal distress	[201, 202]
				rs301435 XM_011518007.2:c.1263-195T>A	T>A, T>C			Intron variant	A significant association was observed only in transmissions to male offspring.	[203]
				rs301979 NM_004170.5:c.1193+88G>A	G>A, G>C/ T/C			Intron variant	Deletion of haplotype associated with early onset OCD	[177]
				rs3087879 XM_011518007.2:c.*1250G>C	G>C			3 Prime UTR variant	G haplotype associated with personal distress G haplotype associated with late-onset OCD	[204]
				rs3780412 XM_011518007.2:c.836+92T>C	T>C / T>G			Intron variant	An allele leads ↑ to hoarding behaviour in woman	[202]
				rs7858819 XM_011518007.2:c.302-1557C>T	C>T			Intron variant	T haplotype associated with ↑ hoarding behaviour	[205]
				rs10491734 XM_047422890.1:c.-152+6922A>T	T>A / T>C / T>G			Intron variant	A haplotype associated with early onset obsessive-compulsive disorder	[206]
23	Vascular monoamine tansporters	SLC18A1 8p21.3	Amygdale Prefrontal brain region Substantia nigra Nucleus accumbens Ventral tegmental area	rs6586896 NM_001135691.2:c.1464+128A>G	T>C, T>G	Solute carrier family 18 member A1	Accumulate cytosolic monoamine into vesicles Storage and release of monoamines Monoaminergic Emotional processing Motivation and reward	Intron variant	Haplotype associated with early onset OCD	[186]
24	Enzymes	MAOA Xp11.3	Limbic system Prefrontal cortex Thalamus Hypothalamus Neocortex	rs1137070 NM_000240.4:c.1410T>C	T>C	Enzyme monoamine oxidases catalyse the oxidation of monoamines like serotonin, dopamine, and adrenaline	Learning and memory Sleep and appetite Consciousness and attention Decision making Working memory	Synonymous Variant	Affect the neurotrophin pathway results ↑ anxiety	[207, 208]
25		MAOB Xp11.3	Striatum Substantia nigra Nucleus accumbens Amygdale Cerebellum Neocortex	rs6651806 XM_005272606.1:c.294+9150T>G	A>C, A>G, A>T	Encoded on the protein Belongs to the flavin monoamine oxidase family catalyse the oxidation of monoamines like serotonin, dopamine, and adrenaline	Emotional processing Motivation and memory consolidation Motor coordination Learning and memory	Intron variant	Haplotype ↑ OCD risk	[209]
				rs1799836 NM_000898.5:c.1348-36A>T	T>A, T>C			Intron variant	Haplotype ↑ OCD risk	[209]
26		COMT 22q11.21	Prefrontal cortex Dorsolateral prefrontal cortex Striatum Nucleus accumbens Substantia nigra	rs362204 XM_006724243.4:c.2782-1531del	delC / dupC / dupCC / insCTG(C)8	COMT is a protein-coding gene Inactivation of catecholamine neurotransmitter and catechol by O-methylation	Reward and motivation Motor control Habit formation and decision-making Attention deficit and executive dysfunctions Dopamine signalling and plasticity	3 Prime UTR Variant	Childhood trauma leads to ↑ risk of developing OCD	[209]
				rs4680 NM_000754.4:c.472G>A	G>A			Missense variant MIR4761: 2KB upstream variant	G allele associated with ↑ anxiety behaviour	[210]
				rs2075507 NM_006440.5:c.103+1132C>T	G>A, G>C, G>T			TXNRD2: Intron variant COMT: 2KB upstream variant	Associated with hoarding and washing symptom ↑ ordering symptoms in male ↑ washing symptoms in female	[211]
27	Other genes	TPH1 11p15.1	Pineal gland Medulla oblongata Raphae nuclei	rs1800532 NM_004179.2:c.803+221C>A	G>T	Protein encoded gene Oxidizes L-tryptophan to 5-hydroxy-l-tryptophan	Role in serotonin synthesis Production of melatonin Control sleep-wake cycle Circadian rhythm Anxiety Depression and Mood disturbances	Intron variant	A and C alleles may be associated with suicidal behaviour	[8]
28		TPH2 12q21.1	Raphe nuclei	rs4570625 NG_008279.1:g.4298G>A	G>A, G>T	Protein encodes gene Rate limiting step in the synthesis of serotonin	Modulate serotonin synthesis Mood regulation Sleep regulation Learning and memory	2KB upstream variant	G allele having ↑ risk of developing OCD	[212]
				rs4565946 XM_005268642.1:c.273+1256C>A	C>A, C>G, C>T			Intron variant	C allele having ↑ risk of developing OCD	[212]
29		DLGAP1 18p11.31	Cortex Hippocampus CA1 and CA2 regions	rs3866988 NM_004746.3:c.1592-28995G>A	C>T	Structural constituent of postsynaptic density Involved in aggresome assembly	Regulation of postsynaptic neurotransmitter activity Regulation of proteasomal protein catabolic process Cognitive flexibility	Intron variant	↑ Anxiety-like behaviour	[213]
30		DLGAP2	Striatum Cerebellum Prefrontal cortex	rs6558484 NM_004745.4:c.1202+17164T>A	T>A, T>C, T>G	Structural constituent of postsynaptic density	Synapse organization and signalling in neuronal cells Movement control Reward processing	Intron variant	Associated with change in brain volume of ACC, OFC, and thalamus in OCD	[214]
				rs7014992 NM_004745.4:c.1202+17987A>C	A>C, A>G, A>T			Intron variant	Associated with change in brain volume of ACC, OFC, and thalamus in OCD	[214]
31		SLITRK5 13q31.2	Prefrontal cortex Hippocampus Striatum	rs9582391 NM_001384609.1:c.-8-1459A>C	A>C / A>G / A>T	SLITRK5 are integral membrane protein	Neurite growth modulation Synaptic development and maturation Neuronal survival and function	Intron variant	↑ Grooming-like behaviour	[208]
32		BDNF 11p14.1	Hippocampus Cortex Basal forebrain	rs6265 NM_170735.6:c.196G>A	C>T	The gene encodes members of nerve growth factors family of proteins	Participate in axonal growth Modulation of dendritic growth and morphology Regulator of synaptic transmission and plasticity	Missense variant	Associated with sexual and religious obsession	[75]

**Abbreviations:** A allele: Adenine allele; ACC: Anterior cingulate cortex; AMPA: α-amino-3-hydroxy-5-methyl-4-isoxazolepropionic acid receptor; BDNF: Brain-derived neurotrophic factor; C allele: Cytosine allele; cAMP: Cyclic adenosine monophosphate; COMT: Catechol-O-methyltransferase; CT genotype: Heterozygous genotype with one C allele and one T allele; DAD3: Dopamine transporter DAT3; DLGAP1: Discs, large homolog 1 (PSD-95); DLGAP2: Discs, large homolog 2 (SAP90/PSD-93); DRD1: Dopamine receptor D1; DRD2: Dopamine receptor D2; DRD3: Dopamine receptor D3; DRD4: Dopamine receptor D4; EAATs: Excitatory amino acid transporters; G allele: Guanine allele; GG genotype: Homozygous genotype with two G alleles; Gi: G protein subunit alpha i (Giα); GRIA1: Glutamate receptor, ionotropic, AMPA 1; GRIK2: Glutamate receptor, ionotropic, kainate 2; GRIK3: Glutamate receptor, ionotropic, kainate 3; GRIN2B: Glutamate receptor, ionotropic, N-methyl-D-aspartate 2B; Gs: G protein subunit alpha s (Gsα); HTR1A: 5-Hydroxytryptamine receptor 1A; HTR1B: 5-Hydroxytryptamine receptor 1B; HTR2A: 5-Hydroxytryptamine receptor 2A; HTR2C: 5-Hydroxytryptamine receptor 2C; HTR3A: 5-Hydroxytryptamine receptor 3A; HTR3B: 5-Hydroxytryptamine receptor 3B; HTR3C: 5-Hydroxytryptamine receptor 3C; HTR3D: 5-Hydroxytryptamine receptor 3D; HTR3E: 5-Hydroxytryptamine receptor 3E; MAOA: Monoamine oxidase A; MAOB: Monoamine oxidase B; NMDA: N-methyl-D-aspartate receptor; OFC: Orbitofrontal cortex; PI3K: Phosphoinositide 3-kinase; SLC18A1: Solute carrier family 18 member 1 (vesicular monoamine transporter); SLC1A1: Solute carrier family 1 member 1 (high-affinity glutamate transporter); SLC6A3: Solute carrier family 6 member 3 (dopamine transporter); SLC6A4: Solute carrier family 6 member 4 (serotonin transporter); SLITRK5: Slit and Trk-like family member 5; SSRI: Selective serotonin reuptake inhibitor; T allele: Thymine allele; TPH1: Tryptophan hydroxylase 1; TPH2: Tryptophan hydroxylase 2.

**Table 2:** Gene-based diagnostic tests for OCD.

**S. No.**	**Test Name**	**Method Name**	**Genes/chromosome Position**	**Name of Laboratory**
1	Genomind pharmacogenetic report	Target variant analysis	BDNF (11p14.1) SLC6A4 (17q11.2) COMT (22q11.21)	Genomind, Inc. Genomimnd, Inc United States
2	Huntington disease repeat expansion analysis (prenatal diagnosis)	Target variant analysis	HTT (4p16.3)	Baylor Genetics United States
3	Huntington disease repeat expansion analysis	Target variant analysis	HTT (4p16.3)	Baylor Genetics United States
4	Methylation analysis for Prader Willi/Angelman syndrome	Methylation analysis	15q11	GENETIX Centro de Investigacion en Genetica Humana y Reproductiva Colombia
5	SLC6A4 - NGS, including CNV analysis	Sequence analysis of the entire coding region	SLC6A4	Centogene AG - the Rare Disease Company
6	Memory impairment, susceptibility to BDNF gene (sequence analysis-all coding exons; postnatal)	Intergen Genetics and Rare Diseases Diagnosis Center Turkey	BDNF (11p14.1)	Intergen Genetics and Rare Diseases Diagnosis Center Turkey
7	Obsessive-compulsive disorder, protection against autosomal-dominant BDNF gene (164230) (sequence analysis-all coding exons; postnatal)	Intergen Genetics and Rare Diseases Diagnosis Center Turkey	BDNF (11p14.1)	Intergen Genetics and Rare Diseases Diagnosis Center Turkey
8	Obsessive-compulsive disorder, susceptibility to autosomal-dominant OCD (HTR2A gene;164230) (sequence analysis-all coding exons; postnatal)	Intergen Genetics and Rare Diseases Diagnosis Center Turkey	HTR2A (13q14.2)	Intergen Genetics and Rare Diseases Diagnosis Center Turkey
9	Obsessive-compulsive disorder, autosomal-dominant SLC6A4 gene (164230) (sequence analysis-all coding exons; postnatal)	Intergen Genetics and Rare Diseases Diagnosis Center Turkey	SLC6A4 (17q11.2)	IIntergen Genetics and Rare Diseases Diagnosis Center Turkey
10	SLC6A4 full gene sequencing analysis	MNG Laboratories (Medical Neurogenetics, LLC.) United States	SLC6A4 (17q11.2)	MNG Laboratories (Medical Neurogenetics, LLC.) United States
11	Neurotransmitter metabolism deficiency NGS panel	Fulgent Genetics United States	HTR1A (5q12.3) HTR1B (6q14.1) HTR1D (1p36.12) HTR2A (13q14.2) HTR2B (2q37.1) HTR2C (Xq23) HTR3A (11q23.2) HTR5A (7q36.2) HTR6 (1p36.13) HTR7 (10q23.31) MAOA (Xp11.3) MAOB (Xp11.3) SLC1A3 (5p13.2) SLC6A3 (5p15.33) SLC6A4 (17q11.2) SLC18A2 (10q25.3)	Fulgent Genetics United States
12	Autism spectrum disorders (Expanded panel), panel massive sequencing (NGS); 77 genes	Sequence analysis of the entire coding region	SLC6A4 (17q11.2) CACNA1C (12p13.33)	Reference Laboratory Genetics Spain
13	Rett syndrome and related disorders, panel massive sequencing (NGS); 18 genes	Sequence analysis of the entire coding region	BDNF (11p14.1)	Reference Laboratory Genetics Spain
14	BDNF gene sequencing and deletion/duplication analysis	Sequence analysis of the entire coding region Deletion/duplication analysis	BDNF (11p14.1)	DDC Clinic Molecular Diagnostics Laboratory DDC Clinic, Center for Special Needs Children United States
15	SLC6A4 single gene	Sequence analysis of the entire coding region Deletion/duplication analysis	SLC6A4 (17q11.2)	Fulgent Genetics United States
16	HTR2A single gene	Sequence analysis of the entire coding region Deletion/duplication analysis	HTR2A (13q14.2)	Fulgent Genetics United States
17	BDNF single gene	Sequence analysis of the entire coding region Deletion/duplication analysis	BDNF (11p14.1)	Fulgent Genetics United States
18	Clinical exome	Sequence analysis of the entire coding region Deletion/duplication analysis	SLC6A3 (5p15.33) SLC6A4 (17q11.2)	Fulgent Genetics United States
19	Serotonin metabolism deficiency NGS panel	Sequence analysis of the entire coding region Deletion/duplication analysis	HTR3E (3q27.1) HTR1A (5q12.3) HTR1B (6q14.1) HTR2A (13q14.2) HTR2B (2q37.1) HTR2C (Xq23) HTR3A (11q23.2) HTR5A (7q36.2) HTR6 (1p36.13) HTR7 (10q23.31) SLC6A4 (17q11.2) TPH1 (11p15.1) HTR3B (11q23.2) HTR3C (3q27.1)	Fulgent Genetics United States
20	Central hypoventilation syndrome NGS panel	Sequence analysis of the entire coding region Deletion/duplication analysis	BDNF (11p14.1) GDNF (5p13.2) ASCL1 (12q23.2) RET (10q11.21)	Fulgent Genetics United States
21	Intellectual disability NGS panel	Sequence analysis of the entire coding region Deletion/duplication analysis	SLC46A1 (17q11.2) MAOA (Xp11.3) BDNF (11p14.1	Fulgent Genetics United States
22	Lung disorders NGS panel	Sequence analysis of the entire coding region Deletion/duplication analysis	BDNF (11p14.1) SCNN1B (16p12.2) SCNN1G (16p12.2) SFTPB (2p11.2) SFTPC (8p21.3)	Fulgent Genetics United States
23	Diabetes-obesity NGS panel	Sequence analysis of the entire coding region Deletion/duplication analysis	SDCCAG8 (1q43-44) TTC8 (14q31.3) BBS5 (2q31.1) PPARGC1B (5q32) ADRB2 (5q32)	Fulgent Genetics United States
24	Autism NGS panel	Sequence analysis of the entire coding region Deletion/duplication analysis	PQBP1 (Xp11.23) LAMC3 (9q34.12) SLC9A6 (Xq26.3) RAI1 (17p11.2)	Fulgent Genetics United States

**Abbreviations:** BDNF: Brain-derived neurotrophic factor; SLC6A4: Solute carrier family 6 member 4 (dopamine transporter); COMT: Catechol-O-methyltransferase; HTT: Huntingtin; HTR2A: 5-Hydroxytryptamine receptor 2A; SLC1A3: Solute carrier family 1 member 3 (glutamate transporter 1); SLC6A3: Solute carrier family 6 member 3 (dopamine transporter); HTR3A: 5-Hydroxytryptamine receptor 3A; HTR1A: 5-Hydroxytryptamine receptor 1A; HTR1B: 5-Hydroxytryptamine receptor 1B; HTR1D: 5-Hydroxytryptamine receptor 1D; HTR2B: 5-Hydroxytryptamine receptor 2B; HTR2C: 5-Hydroxytryptamine receptor 2C; HTR5A: 5-Hydroxytryptamine receptor 5A; HTR6: 5-Hydroxytryptamine receptor 6; HTR7: 5-Hydroxytryptamine receptor 7; MAOA: Monoamine oxidase A; MAOB: Monoamine oxidase B; SLC18A2: Solute carrier family 18 member 2 (vesicular monoamine transporter 2); CACNA1C: Calcium voltage-gated channel subunit alpha1 C; GDNF: Glial cell line-derived neurotrophic factor; ASCL1: Achaete-scute complex homolog 1; RET: Ret proto-oncogene; SLC46A1: Solute carrier family 46 member 1 (prostate-specific membrane antigen); TPH1: Tryptophan hydroxylase 1; HTR3B: 5-Hydroxytryptamine receptor 3B; HTR3C: 5-Hydroxytryptamine receptor 3C; SCNN1B: Sodium/chloride channel, non-neuronal, beta subunit; SCNN1G: Sodium/chloride channel, non-neuronal, gamma subunit; SFTPB: Surfactant protein B; SFTPC: Surfactant protein C; SDCCAG8: Serine/threonine-protein kinase SDCCAG8; TTC8: Tetratricopetide repeat domain 8; BBS5: Bardet-Biedl syndrome 5; PPARGC1B: Peroxisome proliferator-activated receptor gamma coactivator 1 beta; ADRB2: Beta-2 adrenergic receptor; PQBP1: Polyglutamine binding protein 1; LAMC3: Laminin, member 3.

**Table 3:** Clinical trials on various behavioural therapies and brain stimulation therapies and their effect on OCD.

**S. No.**	**Therapy Name**	**Clinical Trial ID**	**Clinical Trial Completion/ ongoing Phase**	**Number of Participants**	**Age Groups**	**Sponsor**	**Key Findings**	**References**
**Behavioural Therapies**								
1	Cognitive behavioural therapy	NCT00680602	Phase 4	158	18 years to 65 years adults and older adults	University of Sao Paulo	Significant reductions in OCD symptoms by Y-BOCS The mean decrease in Y-BOCS was 23.13% Improvement in anxiety and depression symptoms Improved social functioning and quality of life	[267]
2	Behavioural therapy for childhood OCD	NCT00000386	Phase 3	N/A	8 years to 17 years kids and teenagers	National Institute of Mental Health (NIMH)	Reduction in OCD severity compared to PRT 57.1% reduction in CY-BOCS score	[258]
3	Family-based treatment of early childhood OCD	NCT00055068	Phase 3	38	5 years to 8 years children	Rhode Island Hospital	Reduced OCD symptoms 34.4% decrease in CY-BOCS Decreased anxiety and depression symptoms	[257]
4	Brief behavioural treatment for anxiety in young children (PLET)	NCT02051192	Phase 1, phase 2	58	3 years to 7 years children	University of South Florida	PLET decreased anxiety symptoms Improved children’s overall functioning and emotional well-being 90.91% reduction in SCAS score	https://clinicaltrials.gov/study/NCT02051192
5	Internet-based Cognitive Behavior Therapy (CBT) for Obsessive-Compulsive Disorder (OCD)	NCT01347099	Phase 2	101	18 years and older individuals	Karolinska Institutet	Reduction in OCD symptoms 66.7% reduction in Y-BOCS Improvement in anxiety and depression symptoms	[259]
6	Booster as an adjunct to internet-based Cognitive Behavioural Therapy (CBT)	NCT01525576	Phase 2	98	18 years and older adults	Karolinska Institutet	ICBT improved OCD symptoms Improved general functioning	[268]
7	Videophone-administered cognitive-behavioural therapy for pediatric obsessive-compulsive disorder	NCT00881465	Phase 2	32	7-17 years children and teenagers	University of South Florida	Significant decrease in Y-BOCS The percentage reduction of Y-BOCS was greater than the control group Reduced OCD symptoms in children and teenagers	https://clinicaltrials.gov/study/NCT00881465
8	Pilot trial of cognitive and behavioural treatment for compulsive hoarding compared to wait list control	NCT00073346	Phase 1	52	18 years and older adults	Boston University Charles River Campus	Participants (66.7%) in the CBT+MI group achieved clinical significance Higher motivation, self-efficacy, and satisfaction	https://clinicaltrials.gov/study/NCT00073346
9	Cognitive behavioural therapy plus motivational interviewing for the treatment of obsessive-compulsive disorder	NCT00316316	Phase 1	30	18 years to 70 years old adults and older adults	New York State Psychiatric Institute	CBT+MI group exhibited significantly decreased YBOCS compared to CBT alone 23.5 points reduction in YBOCS	[260]
10	Attention training for childhood obsessive-compulsive disorder: an open case series (AMPOCD)	NCT01708226	Phase 1	6	8 years to 17 years children and teenagers	University of California, Los Angeles	A reduction of 26.7 points in Y-BOCS showed improvement (47%) Significance reduction obtained in 50% of participants Reduction in OCD symptoms	https://clinicaltrials.gov/study/NCT01708226
11	Cognitive behavioural therapy for children with autism spectrum disorder and obsessive-compulsive behaviour	NCT03123146	N/A	37	7 years to 13 years children	Tricia Vause	Significant decrease in OCBs measured by Y-BOCS The percentage of reduction in CY-BOCD was higher than the control group Reduced anxiety Improvement in daily functioning, social interaction, and emotional regulation	[269]
12	Task control circuit targets for obsessive compulsive behaviors in children	NCT03584945	N/A	169	6 to 14 years old children	University of Michigan	Cognitive control training reduced OCD symptoms Enhanced cognitive control ability	[270]
13	Risperidone or cognitive-behavioural therapy for improving medication treatment for obsessive-compulsive disorder	NCT00389493	N/A	100	18 years to 70 years adults and older adults	New York State Psychiatric Institute	Patients responded to EX/RP (80%) compared to risperidone (23%) and placebo (15%). EX/RP + SRI reduced OCD symptoms Reduction in Y-BOCS score of about 9.72	[63]
14	CO_2_ reactivity as a biomarker of non-response to exposure-based therapy	NCT05467683	N/A	600	18 years to 70 years adults or older adults	University of Texas at Austin	Proposed to find novel biomarker for predicting non-response to EBT for the treatment of anxiety	[271]
15	Evaluating the effects of a computerized training program coupled with Cognitive Behavioural Therapy (CBT) for OCD	NCT03855943	N/A	100	18 years to 65 years adults or older adults	Hebrew University of Jerusalem	Proposed to decrease anxiety and depression by receiving CBT+CT Improvement in quality of life Expected to improve cognitive flexibility, self-regulatory skills, and attentional control	https://clinicaltrials.gov/study/NCT03855943
16	Internet-based, parent-led cognitive-behavioural therapy for anxiety in youth With ASD	NCT05284435	N/A	256	7 years to 15 years children	Baylor College of Medicine	Proposed to decrease anxiety sensitivity Reduction in repetitive behaviour CBT-E and iCBT-EV were proposed to decrease anxiety	https://clinicaltrials.gov/study/NCT05284435
17	Effect of SC-ICBT on adults with OCD: a three-arm randomized controlled trial	NCT05528224	N/A	114	18 years to 50 years adults	Shanghai Mental Health Center	Proposed to improve treatment adherence and engagement, form cost-effective treatment, and reduce the mental health burden	https://clinicaltrials.gov/study/NCT05528224
18	Efficacy of augmentation of cognitive behavioural therapy with transcranial direct current stimulation for Obsessive-Compulsive Disorder (OCD)	NCT04527302	N/A	60	18 years to 50 years adults	Shanghai Mental Health Center	Proposed to reduce OCD symptoms by combination of CBT+tDCS, develop more effective and personalized treatment, and lower the level of stress and burden	https://clinicaltrials.gov/study/NCT04527302
19	Family involvement in CBGT of OCD: a randomized controlled trial	NCT04071990	N/A	80	18 years to 65 years adults and older adults	University Hospital, Ghent	A significant reduction in OCD symptoms by combining CBGT+FI Lower level of stress and burden Improved family functioning Enhanced overall well-being	https://clinicaltrials.gov/study/NCT04071990
**Brain Stimulation**								
1	Deep brain stimulation for Obsessive Compulsive Disorder (OCD PMCF)	NCT01135745	Phase 4	32	18 years and older adults	MedtronicNeuro	DBS reduced OCD symptoms measured by YBOCS Long-term DBS was observed as beneficial	[266]
2	Comparison of DBS targets in obsessive-compulsive disorder (PRESTOC2)	NCT01807403	Phase 3	8	18 years to 60 years adults	Assistance Publique - Hôpitaux de Paris	Reduction in OCD symptoms Valuable in individuals with severe and resistant OCD	[272]
3	Neurocircuitry of obsessive-compulsive disorder: modulation by transcranial magnetic stimulation	NCT02704117	Phase 3	19	18 years to 70 years adults and older adults	Butler Hospital	Real TMS significantly resulted in changes in brain activity compared to sham TMS Decreased OCD symptom severity	https://clinicaltrials.gov/study/NCT02704117
4	TMS for Exposure Therapy Resistant OCD (TETRO)	NCT05331937	Phase 3	250	18 years adults and older adults	Amsterdam UMC, location VUmc	Decreased OCD symptoms by rTMS Evaluation of the safety and tolerability of rTMS	https://clinicaltrials.gov/study/NCT05331937
5	Deep Brain Stimulation (DBS) for the treatment of refractory Obsessive-Compulsive Disorder (OCD)	NCT04217408	Phase 2	10	18 years to 70 years adults and older adults	Sunnybrook Health Sciences Centre	Decreases OCD symptoms measured by YBOCS Measured brain connectivity and functions	https://clinicaltrials.gov/study/NCT04217408
6	Subthalamic Nucleus (STN) stimulation and Obsessive-Compulsive Disorder (OCD)	NCT00169377	Phase 1, phase 2	16	18 years to 60 years adults and older adults	Marie-laure Welter	Potential decrease in OCD symptoms as assessed by Y-BOCS in comparison to sham stimulation	[273]
7	Refining the target for Deep Brain Stimulation (DBS) in severe, treatment-refractory Obsessive Compulsive Disorder (OCD)	NCT01985815	Phase 1	6	18 years to 65 years adults and older adults	Universitaire Ziekenhuizen KU Leuven	Potential decrease in OCD symptoms as assessed by Y-BOCS	https://clinicaltrials.gov/study/NCT01985815
8	Efficacy study of transcranial magnetic stimulation for treatment of obsessive-compulsive disorder	NCT01540305	Phase 2	22	18 years to 60 years adults and older adults	University of Brasilia	rTMS significantly reduced OCD symptoms A decrease in the YBOCS score indicated a reduction in symptoms rTMS modulated DLPFC and regulated mood and anxiety	https://clinicaltrials.gov/study/NCT01540305
9	Study on repetitive transcranial magnetic stimulation’s efficacy for Obsessive Compulsive Disorder (OCD) (TMS-TOC)	NCT00822601	Phase 2	40	18 years to 65 years adults and older adults	Assistance Publique - Hôpitaux de Paris	rTMS significantly reduced OCD symptoms A decrease in the YBOCS score indicated a reduction in symptoms rTMS modulated DLPFC and regulated mood and anxiety	https://clinicaltrials.gov/study/NCT00822601
10	rTMS over the supplementary motor area for treatment-resistant Obsessive-Compulsive Disorder (rTMSOCD)	NCT03211221	Phase1, phase 2	30	18 years to 65 years adults and older adults	CNS Onlus	Decrease OCD symptoms assessed by YBOCS Evaluation of changes in mood and anxiety by BDI and HAM-A	https://clinicaltrials.gov/study/NCT03211221

**Abbreviations:** AMPOCD: Attention Training for Childhood Obsessive Compulsive Disorder; BDI: Beck Depression Inventory; CBT: Cognitive behavioural therapy; CBT-E: Cognitive behavioural therapy for eating disorders; CY-BOCS: Children's Yale-Brown Obsessive Compulsive Scale; DBS: Deep brain stimulation; DLPFC: Dorsolateral prefrontal cortex; EBT: Exposure-based therapy; EX/RP: Exposure and response prevention; FI: Family involvement ; HAM-A: Hamilton Anxiety Rating Scale; ICBT: Internet-based cognitive behavioural therapy; iCBT-EV: Internet-based cognitive-behavioural therapy for eating disorders; NCT: ClinicalTrials.gov Identifier (National Clinical Trial); OCBs: Obsessive-compulsive behaviors; PLET: Parent-led exposure therapy; PMCF: Post-market clinical follow-up; PRT: Prolonged exposure and response prevention therapy; rTMS: Repetitive transcranial magnetic stimulation; SRI: Serotonin reuptake inhibitors; TMS: Transcranial magnetic stimulation; TMS-TOC:Transcranial magnetic stimulation’s efficacy on obsessive compulsive disorder; Y-BOCS: Yale-Brown Obsessive Compulsive Scale.

**Table 4:** Ongoing and completed clinical trials on various therapeutic agents for OCD.

**S. No.**	**Class**	**Compound/ drug Name**	**Clinical Trial ID**	**Study Status**	**Gene Involved/ target involved/ Mechanism**	**Dose and Route**	**No. of Individuals**	**Key Findings**	**References**
**Recently Completed Clinical Trails**									
1	Cannabinoids	Nabilone Nabilone + EX/RP	NCT02911324	Phase 1 Phase 2 completed	• Cannabinoid 1 receptor agonist • Effect on neurocognitive process • Threat response, processing of fear signals, and habitual behavior	1 mg daily (BID) over 4 weeks	6 (Nabilone) 5 (Nabilone + EX/RP) Total adults (11)	• Enhance fear extinction learning • Modulate CSTC circuit • Reducing repetitive behaviour	[275]
2	Dopamine agents	Tolcapone	NCT03348930	Phase 2 Phase 3 completed	• Tolcapone inhibited COMT • Enhanced cortical dopamine transmission • Controlled habitual patterns of behavior	200 mg or 100 mg twice daily	20 adults	• Significant symptomatic improvement in OCD	[292]
3		Methylphenidate hydrochloride	NCT02194075	Phase 4 completed	• DAT and NET reuptake inhibition	100-300 mg/day for 8 weeks	60 adults	• Improvement in Y-BOCS obsession subscale score and • HAMA score comparison to control group	[276]
4		Pramipexole, amisulpride	NCT00471588	Phase 1	• Dopamine D1/D2 receptor agonist and antagonist	1.5mg single dose 400mg single dose	52 adults and older adults	• Pramipexole increases YBOCD scores, worsening of OCD symptoms • Amisulpride reduced YBOCS scores	https://clinicaltrials.gov/study/NCT00471588
5	Glutamate agents	Minocycline	NCT01695291	Phase 2	• Glutamate modulating activity (enhancing glial glutamate transport)	2mg/kg/day	31 children and adults	• Significantly improvement in OCD symptoms by Y-BOCS • Improve hoarding symptoms	[293]
6		Topiramate	NCT00182520	Phase 4	• Topiramate may block the Na+ channel • Act as GABA agonist	• 25 mg - 400 mg/day x 12 weeks	23 adult	• Significant improvement in OCD symptoms on Y-BOCS • average decrease in YBOCS score was 26.7%	[294] al., 2010
7	-	Pregabalin	NCT00994786	Phase 4	• Modulation of Ca^2+^ channels • Enhanced GABA activity • Inhibited glutamate release	75mg/day – maximum 600mg/day	15 (adult, older adult)	• Decrese in Y-BOCS score (15%) • Significant improvement in OCD symptoms • Improvement in BDI and BAI scores	https://clinicaltrials.gov/study/NCT00994786
8	Serotonin agents	Ondansetron	NCT00796497	Phase 4 completed	• Indirect inhibitor of cortical-mesolimbic dopamine release	0.25 mg twice daily for 6 weeks	14 adults	• Whole group experiences a 23.2% average decrease in YBOCS-rated symptoms	[277]
9		Ondansetron	NCT03239210	Phase 4 completed	• Selective 5-HT3 (serotonin) receptor antagonist	24 mg/day	110 adult	• Improvement in OCD symptoms • improvement in BDI, BAI, and GAF. • Changes in brain activity in the insula and sensory-motor cortex	[274]
10		Sertraline	NCT03993535	Phase 4	• Selective serotonin reuptake inhibitor	50 mg/day/week- 200 mg/day	250 adults	• Ongoing measurement of severity by Y-BOCS and • Improvement by Clinical Global Impression Scale	https://clinicaltrials.gov/study/NCT03993535
11		Sertraline	NCT02797808	Phase 1 Phase 2	• Selective serotonin reuptake inhibitor	-	41 children	• Significant improvement in striatal connectivity of two groups • Increase in connectivity in the left putamen circuit was significantly correlated with • Clinical improvement on CY-BOCS	[295]
12		Minocycline	NCT01695291	Phase 2	• Act as an antioxidant and reduce inflammation • Helps to alleviate OCD symptoms	2 mg/kg/day for 12 weeks	31 children and adults	• Significant improvement in OCD symptoms in Y-BOCS	https://clinicaltrials.gov/study/NCT01695291
13	Norepinephrine dopamine agents	Methylphenidate ER	NCT01100268	Phase 2 completed	• DAT and NET reuptake inhibition	18mg/day, increasing 18mg per week up to 72mg/day	4 adults	• Decrease hoarding behaviour	[295, 296]
14	Dopamine serotonin norepinephrine	Risperidone	NCT00389493	Phase 4 completed	• D2 and 5HT2A receptor antagonist	0.5mg-4mg/day	100 adults or older adults	• Resperidone + EX/RP had a superior effect on OCD	[63]
15	-	N-acetyl cysteine	NCT01555970	Phase 2	• Modulation of AMPA and NMDA receptors through the cystine-glutamate antiporter	3000 mg/day	40 adults	• Significant decrease in Y-BOCS • Decrease anxiety symptoms	[278]
16	Probiotics/nutraceuticals	*Lactobacillus helveticus* R0052 and *Bifidobacterium longum* R0175	NCT02334644	Phase 4 completed	• Modulation of the immune system	12-week treatment	15 adults and older adults	• Probiotic treatment did not significantly decrease OCD symptoms	https://clinicaltrials.gov/study/NCT02334644
**Ongoing Clinical Trials**									
17	Dopamine agents	Tolcapone	NCT05624528	Phase 2	• Proposed mechanism by acting on the dopamine system • Control cognitive dysfunction • Improve executive function, including cognitive flexibility	100mg twice daily for 2 weeks, then 200mg twice daily for the remaining 6 weeks	85 (adults, older adults)	• Proposed to decrease in obsessive compulsive symptoms on Y-BOCS significantly	[292]
18	Cannabinoids	Nabilone	NCT04880278	Phase 1 Phase 2	• Cannabinoid 1 receptor agonist • Effect on neurocognitive process • Threat response, processing of fear signals, and habitual behavior	1 mg orally	60 adults	• Proposed to check OCD symptoms by Y-BOCS and	[275]
19		Dronabinol	NCT01093976	Phase 2	• CB1/CB2 receptor agonist	2.5 mg/day for week 3 5mg/day for week 6 10 mg/day for week 9	14 adult and older adults	• Proposed to check OCD symptoms by Y-BOCS and • Side effect • Check mood, anxiety • Cognition and quality of life	https://clinicaltrials.gov/study/NCT01093976
20		Epidiolex	NCT04978428	Phase 2	• Interaction with cannabidiol receptors • Having anticonvulsant action	2.5 mg/kg and 5 mg/kg	15 (adults and older adults)	• Proposed to decrease the obsessive-compulsive related symptoms	[275]https://clinicaltrials.gov/study/NCT04978428
21	Serotonin agents	Psilocybin	NCT05546658	Early phase 1	• Serotonergic psychedelic • 5-HT(1A) and 5-HT(2A/2C) • Glutamatergic modulation • Anti-inflammatory effect	(20mg in the first session, if tolerated then 30mg)	30 (adults and older adults)	• Proposed to check OCD symptoms by Y-BOCS and • To Check mood, anxiety • Cognition and quality of life	https://clinicaltrials.gov/study/NCT05546658
22		Psilocybin	NCT05370911	Phase 1	• Serotonergic psychedelic • 5-HT(1A) and 5-HT(2A/2C) • Glutamatergic modulation • Anti-inflammatory effect	(25 mg first dose and second dose 25/30 mg	30 (adults or older adults)	• Proposed to check OCD symptoms by Y-BOCS and • To Check mood, anxiety • Cognition and quality of life	https://clinicaltrials.gov/study/NCT05370911
23		Psilocybin	NCT03300947	Phase 1	• Serotonergic psychedelic • 5-HT(1A) and 5-HT(2A/2C) • Glutamatergic modulation • Anti-inflammatory effect	100 mcg/kg 300 mcg/kg	15 (adults and older adults)	• Proposed to check OCD symptoms by Y-BOCS • Side effect • Check mood, anxiety • Cognition and quality of life	https://clinicaltrials.gov/study/NCT03300947
24		Psilocybin	NCT03356483	Phase 1	• Serotonergic psychedelic • 5-HT(1A) and 5-HT(2A/2C) • Glutamatergic modulation • Anti-inflammatory effect	0.25 mg/kg	30 adults	• Proposed to check OCD symptoms by Y-BOCS and • Neuroimaging data at baseline	[242]
25	-	Sertraline	NCT04539951	Phase 2	• Selective serotonin reuptake inhibitor • Inhibition of CNS neuronal uptake of serotonin (5HT)	200mg and 300mg	1600 adults	• Proposed to measure improvement in OCD symptoms by Y-BOCS	[270]
26		Sertraline + fluvoxamine	NCT04963257	Phase 4	• Selective serotonin reuptake inhibitor • Inhibition of CNS neuronal uptake of serotonin (5HT)	150 mg/day	400 child- adults	• Proposed to check decrease in obsessive-compulsive symptoms by Y-BOCS	https://clinicaltrials.gov/study/NCT04963257
27	-	Escitalopram	NCT04336228	Phase 4	• Selective serotonin reuptake inhibitor by binding on orthosteric binding site of Serotonin Transporter (SERT)	20mg daily for 3-4 weeks	48 adults	• Proposed to check the status of central serotonin 5-HT in compulsive behaviour	https://clinicaltrials.gov/study/NCT04336228
28	Glutamate agents	Nitrous oxide gas	NCT03826693	Phase 2	• Glutamate AMPA and NMDA receptors antagonist • Low voltage calcium channel inhibitor	50% oxygen/50% nitrous Oxide	45 (adults and older adults)	• Proposed to decrease the Obsessive-compulsive symptoms on Y-BOCS	[297]
29	-	Troriluzole	NCT03299166	Phase 2 Phase 3	• Glutamate transporter modulation • NMDA glutamate receptor antagonism	140 mg capsule for 4 weeks and 200mg (140 mg + 60mg) for an additional 8 weeks once a day	426 (adults and older adults)	• Adjunctive therapy showed improvement over placebo • Primary outcome significant at week 8 (*p*= 0.05)	[298]
30	-	Riluzole	NCT00523718	Phase 2	• NMDA glutamate receptor antagonism • Inhibiting voltage-gated sodium channels	50 mg PO bid, 12 weeks	40 (adults and older adults)	• Greater Y-BOCS improvement in the riluzole group compared to placebo • In outpatient, at least a partial 25% improvement in riluzole than placebo	[299]
31	-	N-acetyl cysteine	NCT01172275	Phase 2	• Modulation of AMPA and NMDA receptors through the cystine-glutamate antiporter	1900mg OD× 1 week 1900mg BD×1 week 1900mg TD×10 weeks	11 child	• Safe and well-tolerated in children • No significant difference between N-acetyl cysteine and placebo in reducing OCD symptoms	[300]
32		Ketamine	NCT01100255	Phase 2 completed	• Noncompetitive antagonist of *N*-methyl-d-aspartate (NMDA)	0.5mg/kg IV over 40 minutes	15 adults	• Significant improvement in obsessions compared to placebo	[301]
33	-	Ketamine	NCT05565352	NA	• Noncompetitive antagonist of *N*-methyl-d-aspartate (NMDA)	0.5mg/kg IV over 40 minutes for 4 weeks 2.0mg/kg, 2.5mg/kg twice weekly over 4 months	140 (adults and older adults)	• Proposed to check improvement in OCD symptoms by Y-BOCS • Proposed improvement in mood and anxiety	[302]
34		Ketamine	NCT05577585	Phase 3	• Noncompetitive antagonist of *N*-methyl-d-aspartate (NMDA)	05 mg/kg iv infusion	30 adults and older adults	• Proposed to check improvement in OCD symptoms by Y-BOCS	https://clinicaltrials.gov/study/NCT05577585
35	-	Dextromethorphan	NCT05565352	Phase 2	• Non-selective serotonin reuptake inhibitor • Sigma-1 receptor agonist	15 mg-60 mg per dose twice daily	60 adults	• Proposed to check improvement in OCD symptoms by Y-BOCS • Improvement in clinical insight by BABS	https://clinicaltrials.gov/study/NCT05565352
36	Immunomodulator and anti-inflammatory effects	Naproxen sodium (for PANDAS/PANS)	NCT04015596	Phase 4	• Nonselective COX-1 and COX-2 inhibitor	(10mg/kg, by mouth, twice a day)	70 children	Proposed to check the OCD severity by • CY-BOCS II and CY-BOCS I • Change in c –c-reactive protein • Erythrocyte sedimentation rate	[279]
37		Rituximab(for PANDAS/PANS)	NCT03983031	Phase 1 completed	• Antibodies against CD20, cluster of differentiation	1000 mg	11 adults	• There is a slight decrease in the ocd symptoms by YB-OCS and PSP	[280]
38		Rituximab	NCT04323566	Phase 2	• Antibodies against CD20, cluster of differentiation	500 mg	40 adults	• Proposed to check improvement in OCD symptoms by Y-BOCS	https://clinicaltrials.gov/study/NCT04323566
39		Celecoxib	NCT04786548	Phase 2	• COX-2 inhibitor	100 mg twice daily, increased dose to 200 mg	21 adults	• To measure the change in OCD symptoms from baseline to endpoint as measured by the	[281]
40	-	Celecoxib	NCT04673578	Phase 2	• COX-2 inhibitor	50 mg orally or 100 mg orally twice daily	80 children	• Proposed to check the severity proportion of patients in each group of OCD by CY-BOCS	[281]

**Abbreviations:** AMPA: Alpha-amino-3-hydroxy-5-methyl-4-isoxazolepropionic acid; AMPK: AMP-activated Protein Kinase; BABS: Brown Assessment of Beliefs Scale; BAI: Beck Anxiety Inventory; BDI: Beck Depression Inventory; BID: Twice a day; CB1: Cannabinoid 1 receptor; CD20: Cluster of differentiation 20; COMT: Catechol-O-methyltransferase; COX: Cyclooxygenase; CSTC: Cortico-striato-thalamo-cortical; CY-BOCS: Children's Yale-Brown Obsessive Compulsive Scale; DAT: Dopamine transporter; ER: Extended release; EX/RP: Exposure and response prevention; GABA: Gamma-aminobutyric acid; GAF: Global Assessment of Functioning; IV: Intravenous; kg: kilograms; mcg: micrograms; mg: milligrams; NCT: ClinicalTrials.gov Identifier (National Clinical Trial); NET: Norepinephrine transporter; NMDA: N-methyl-D-aspartate; OCD: Obsessive-compulsive disorder; PANDAS: Pediatric autoimmune neuropsychiatric disorders associated with streptococcal infections; PANS: Pediatric acute-onset neuropsychiatric syndrome; PO: Per oral; QD: Once a day; Y-BOCS: Yale-Brown Obsessive Compulsive Scale.

**Table 5:** Various gene-based animal models and associated behaviour in OCD.

Sr. No.	Gene/ receptor	Animal Species	Animal Model	Parameters Involved	Instrument Used	Key Findings in OCD	References
1	Sapap3	Mice	Sapap3 Knockout mice (KO)	Marble burring repetitive self-grooming Head twitches Compulsive checking Gene expression Neuronal morphology Corticostriatal synaptic function	Open field Elevated zero maze Dark light emergence Quantitative real-time PCR Western blotting Electrophysiology Golgi staining	↓ EPSCs in striatal MSNs ↑ Intrinsic excitability ↑ Marble burying behaviour ↑ Self-grooming ↓ Sapap3 mRNA protein expression in OCD All showed a potential role in OCD	[42]
2	HoxB8	Mice	HoxB8 overexpression	Grooming behaviour Bone marrow transplantation Microglial cell analysis Gene expression analysis	Flow cytometer Immunofluorescence microscope Quantitative real-time PCR Microarray platform	Abnormal grooming patterns ↑ Grooming behaviour ↑ Number of activated microglia in the brain All of these altering gene expression	[85, 220]
3	Dlx5/6	Mice	Dlx5/6 double Knockout (KO) model	Dlx5/6 expression levels Adult Parvalbumin (PV) neuronal density Anxiety-like behaviour Compulsive-like behaviour	Elevated plus maze Open field test Marble burying test Nest building test Quantitative real-time PCR Immunohistochemistry	Dlx5/6 expression decreased PV neuronal plasticity Effect on the neuronal plasticity of the prelimbic cortex and CA1 region of the hippocampus ↑ Anxiety-like behaviour ↑ Marble burring behaviour	[219]
4	5-HT2A	Rat	5-HT2A receptor overexpression	Marble burying behaviour Nest building Grooming behaviour Locomotor activity Anxiety-like behaviour *Lactobacillus casei* consumption	Elevated plus maze Open field test Automated marble burring chambers Quantitative Polymerase Chain Reaction (qPCR)	5-HT2A ↑ Marble-buring behaviour ↑ Obsessive-compulsive symptoms *L. casei* treatment was beneficial in all these behaviours	[316]
5	SLITRK5	Mice	SLITRK5-KO mice	Compulsive-like behaviour Marble burying behaviour Self-injurious grooming Anxiety behaviour D1 and D2 dopamine receptor expressions in the striatum Glutamate receptor composition in the striatum	Open field Elevated plus maze Western blotting Quantitative real-time PCR	↓ D1 and ↑ D2 receptor expressions in striatum Altered glutamate receptor composition ↑ Neuronal activity in OFC Excessive grooming ↑ Anxiety-like behaviour ↑ Marble burying	[317]
6	SLITRK5	Mice	SLITRK5-deficient mice	Basal ganglia activity Marble burying behaviour Head twitches Self-grooming Gene expression	IEG immunohistochemistry Electrophysiology Quantitative real-time PCR	↑ Basal ganglia activity in the striatum ↑ Marble burying behaviour ↑ Head twitches, self-grooming SLITRK5 mRNA and protein levels ↓ in the striatum	[317]
7	HT2RC	Mice	5-HT2C-R-KO mice	Increased head dipping Non-nutritive chewing Reduced anxiety	Open field Elevated plus maze Mirror chamber Novel object	5-HT2C receptor KO mouse exhibited multiple compulsive-like behaviours ↑ Excessive chewing The involvement of 5-HT2C receptor in OCD Potential therapeutic target for OCD	[318, 319]
8	SLC1A1/EAAC1	Mice	EAAC1-KO	Marble burring Head twitches Self-grooming Immediate Early Gene (IEG) expression in the striatum (c-Fos) *Ex vivo* intracellular recordings from striatal medium spiny neurons	Open field Quantitative real-time PCR Electrophysiology IEG Immunohistochemistry	EAAC1-KO ↑ increased basal ganglia activity in striatum ↑ Marble burying ↑ Head twitches ↑ Self-grooming ↓ SLC1A1/EAAT3 mRNA levels Potential therapeutic target for OCD	[320]

**Abbreviations:** 5-HT2A: Serotonin 2A receptor; CA1: Cornu Ammonis 1c-Fos: Cellular oncogene fos; Dlx5/6: Distal-less homeobox 5/6; EPSCs: Excitatory postsynaptic currents; HoxB8: Homeobox B8; HT2RC: 5-HT2C receptor; IEG: Immediate early gene; KO: Knockout; MSNs: Medium spiny neurons; OFC: Orbitofrontal cortex; PCR: Polymerase chain reaction; PV: Parvalbumin; Sapap3: Synapse-associated protein 3; SLC1A1/EAAC1: Solute carrier family 1 member 1/excitatory amino acid carrier 1; SLITRK5: SLIT and NTRK-like protein 5.

## LIST OF ABBREVIATIONS

5-HT: Serotonin (5-Hydroxytryptamine)
5-HT1A: Serotonin receptor 1A
5-HT1B: Serotonin receptor 1B
5-HT1D: Serotonin receptor 1D
5-HT1E: Serotonin receptor 1E
5-HT1F: Serotonin receptor 1F
5-HT2A: Serotonin receptor 2A
5-HT2B: Serotonin receptor 2B
5-HT2C: Serotonin receptor 2C
5-HT3: Serotonin receptor 3
5-HT4: Serotonin receptor 4
5-HT6: Serotonin receptor 6
5-HT7: Serotonin receptor 7
5-HTTLPR: Serotonin transporter gene promoter region
5HTT: Serotonin transporter
ABCA1: ATP-binding cassette subfamily A member 1
AC: Adenylyl cyclase
ACC: Anterior cingulate cortex
ACE: Angiotensin-converting enzyme
Ach: Acetylcholine
AD: Alzheimer's disease
ADAM10: A disintegrin and metalloproteinase domain 10
ADAMTS1A: Disintegrin and metalloproteinase with thrombospondin motifs 1
ADHD: Attention deficit hyperactivity disorder
Akt: Protein kinase B
ALX4: ALX homeobox 4
AMD: Age-related macular degeneration
AMPA: Alpha-amino-3-hydroxy-5-methyl-4-isoxazolepropionic acid
ANK3: Ankyrin 3
ANKK1: Ankyrin repeat and kinase domain containing 1
AnVIL: Analysis, visualization, and informatics lab-space
APA: American Psychiatric Association
APOA5: Apolipoprotein A5
APOE: Apolipoprotein E
APOM: Apolipoprotein M
APP: Amyloid precursor protein
AS3MT: Arsenic (+3 oxidation state) methyltransferase
ASD: Autism spectrum disorder
ASXL3: Additional sex combs-like 3
AT: Attention training
ATG16L1: Autophagy related 16-like 1
Aβ: Beta-amyloid
BAF: BAF chromatin remodeling complex
BAI: Beck Anxiety Inventory
BDI: Beck Depression Inventory
BDKRB2: Bradykinin receptor B2
BDNF: Brain-derived neurotrophic factor
Bmal1: Brain and muscle ARNT-like 1
BORCS8: BLOC-1-related complex subunit 8
BPD: Bipolar disorder
BTBD3: BTB domain containing 3
CA: Coronary artery
CACNA1C: Calcium voltage-gated channel subunit alpha1 C
CACNB2: Calcium voltage-gated channel auxiliary subunit beta 2
CAD: Coronary artery disease
CADPS: Calcium-dependent secretion activator
CAMKII: Calcium/calmodulin-dependent protein kinase II
cAMP: Cyclic adenosine monophosphate
CBT: Cognitive behavioural therapy
CD: Crohn's disease
CDH2: Neuronal cadherin
CDI: Children's Depression Inventory
CDKAL1: CDK5 regulatory subunit associated protein 1-like 1
CDKAL1: CDK5 regulatory subunit associated protein 1-like 1
CDKN2A: Cyclin-dependent kinase inhibitor 2A
CDKN2A: Cyclin-dependent kinase inhibitor 2A
CDKN2B: Cyclin-dependent kinase inhibitor 2B
CFH: Complement factor H
CHD8: Chromodomain helicase DNA binding protein 8
CHRNA4: Cholinergic receptor nicotinic alpha 4
CHRNE: Cholinergic receptor nicotinic epsilon subunit
CI: Confidence interval
CLOCK: Circadian locomotor output cycles kaput
CNS: Central nervous system
CNV: Copy number variation
COBRA-FISH: Combinatorial oligonucleotide-based repetitive fluorescence *in situ* hybridization
COMT: Catechol-O-methyltransferase
COX: Cyclooxygenase
CREB: cAMP response element-binding protein
CRISPR-Cas: Clustered regularly interspaced short palindromic repeats and CRISPR-associated protein
CRY1: Cryptochrome 1
CSTC: Cortico-striatal-thalamic-cortical circuit
CTLA-4: Cytotoxic T lymphocyte associated protein 4
CTQ: Childhood Trauma Questionnaire
CTTNBP2: Cortactin binding protein 2
CYP: Cytochrome P450
CYP7A1: Cytochrome P450 family 7 subfamily A member 1
D3 receptors: Dopamine D3 receptors
D4 receptors: Dopamine D4 receptors
D5 receptors: Dopamine D5 receptors
DAOA: D-amino acid oxidase activator
DAT: Dopamine transporter gene (SLC6A3)
DAT1: Dopamine transporter 1
dbGaP: Database of Genotypes and Phenotypes
DBH: Dopamine β-hydroxylase
Dbp: D-Box binding PAR BZIP
DBS: Deep brain stimulation
dCaud: Dorsal portion of caudate nucleus
DDX21: DEAD-box helicase 21
Dec1: Differentiated embryo chondrocyte 1
Dec2: Differentiated embryo chondrocyte 2 (part of the Bhlhb2/Bhlhb3 family)
DISC1: Disrupted in schizophrenia 1
DLGAP: Discs, large homolog-associated protein
DLGAP1: Discs large homolog-associated protein 1
DLGAP2: DLG-associated protein 2
dlPFC: Dorsolateral prefrontal cortex
DNA: Deoxyribonucleic acid
DRD1/DRD2: Dopamine receptor D1/dopamine receptor D2
DRD2: Dopamine D(2) receptor
DRD3: Dopamine D(3) receptor
DRD4: Dopamine D(4) receptor
DRD5: Dopamine D5 receptor gene
DRN: Dorsal raphe nucleus
DSM-5: Diagnostic and Statistical Manual of Mental Disorders, 5^th^ Edition
DSM-III-R: Diagnostic and Statistical Manual of Mental Disorders, Third Edition, Revised
DSM-IV: Diagnostic and Statistical Manual of Mental Disorders, Fourth Edition
DTNBP1: Dystrobrevin binding protein 1
DX: Dizygotic
E4bp4: E4 promoter-binding protein 4 (NFIL3)
EAAT: Excitatory amino acid transporter
EML2: Echinoderm microtubule-associated protein-like 2
ERK: Extracellular regulated protein kinase
ERP: Exposure and response prevention
EX/RP: Exposure and response prevention (often used in the context of cognitive behavioural therapy)
EXT2: Exostosin glycosyltransferase 2
EXT2: Exostosin glycosyltransferase 2
FBL: Fibrillarin
FCBT: Family-based cognitive behavioural therapy
FKBP5: FK506 binding protein 5
FMRI: Functional magnetic resonance imaging
FOIA: Freedom of Information Act
FOXP1: Forkhead box P1
FOXP2: Forkhead box P2
FTO: Fat mass and obesity-associated protein
G × E: Gene-environment interaction
GABA: Gamma-aminobutyric acid
GABA: Gamma-aminobutyric acid
GABA: γ-aminobutyric acid
GABRB2: Gamma-aminobutyric acid type A receptor subunit beta 2
GABRB4: Gamma-aminobutyric acid type A receptor subunit beta 4
GAD1: Glutamate decarboxylase 1
GAD2: Glutamate decarboxylase 2
GAF: Global Assessment of Functioning
Gi-type GPCRs: G-protein-coupled receptors of the inhibitory G protein type
Gi/o: Inhibitory G protein
GINA: Genetic Information and Nondiscrimination Act
GLRB: Glycine receptor beta
GPCR: G protein-coupled receptor
GPi: Globus pallidus internal segment
GRIA: Glutamate ionotropic receptor AMPA type
GRIA1: Glutamate ionotropic receptor AMPA type subunit 1
GRIK: Glutamate ionotropic receptor kainate type
GRIN: Glutamate ionotropic receptor NMDA type
GRIN2B: Glutamate ionotropic receptor NMDA type subunit 2B
GRIP1: Glutamate receptor interacting protein 1
GRM3: Glutamate metabotropic receptor 3
Gs: Stimulatory G protein
Gs-type GPCR: G-protein-coupled receptor of the stimulatory G protein type
GWA: Genome-wide association
GWAS: Genome-wide association study
Gβγ: G beta gamma subunits
HHEX: Hematopoietically expressed homeobox
HLF: Hepatic leukemia factor
HTR: Serotonin receptor
HTR1B: 5-hydroxytryptamine receptor 1B
HTR2A: 5-hydroxytryptamine receptor 2A
IBD: Inflammatory bowel disease
ICBT: Internet-based cognitive behavioural therapy
ICD-11: International Classification of Diseases, 11th Revision
IDDM: Insulin-dependent diabetes mellitus
IDE: Insulin-degrading enzyme
IGF: Inferior frontal gyrus
IGF2BP2: Insulin-like growth factor 2 mRNA-binding protein 2
iGluR: Ionotropic glutamate receptor
IL-12: Interleukin-12
IL-12/IL-23: Interleukin-12/interleukin-23
IL23R: Interleukin 23 receptor
IQCK: IQ motif containing K
ITIH3: Inter-alpha-trypsin inhibitor heavy chain 3
ITIH3-ITIH4: Inter-alpha-trypsin inhibitor heavy chain H3-inter-alpha-trypsin inhibitor heavy chain H4
KCNJ11: Potassium inwardly rectifying channel subfamily J member 11
KDM3B: Lysine demethylase 3B
KIF11: Kinesin family member 11
KO: Knockout
LD: Linkage disequilibrium
LGICs: Ligand-gated ion channels
LTA: Lymphotoxin-α
LTP: Long-term potentiation
MACROD2: MACRO domain-containing 2
MAF: Minor allele frequency
MAO: Monoamine oxidase
MAOA: Monoamine oxidase A
MAOB: Monoamine oxidase B
MAOI: Monoamine oxidase inhibitor
MAPK: Mitogen-activated protein kinase
MDD: Major depressive disorder
MEF2BNB: Muscle-specific MEF2 binding protein
MEF2BNB: Myocyte enhancer factor 2B neighbor
mGluR: Metabotropic glutamate receptor
MHC: Major histocompatibility complex
MI: Motivational interviewing
MIR137: MicroRNA 137
MOG: Myelin oligodendrocyte glycoprotein
mPFC: Medial prefrontal cortex
MPS: Massively parallel sequencing
MR: Mendelian randomization
MRN: Median raphe nuclei
MRS: Magnetic resonance spectroscopy
MZ: Monozygotic
NAA15: N-alpha-acetyltransferase 15
NAcc: Nucleus accumbens
NCAM1: Neural cell adhesion molecule 1
NCT: ClinicalTrials.gov Identifier (National Clinical Trial)
NDUFAF7: NADH dehydrogenase (ubiquinone) complex assembly factor 7
NEGR1: Neuronal growth regulator 1
NGFR: Nerve growth factor receptor
NGS: Next-generation sequencing
NGT: Normal glucose-tolerant
NHGI: National Human Genome Institute
NLGN1: Neuroligin 1
NMDA: N-methyl-D-aspartate
NOS1AP: Nitric oxide synthase 1 adaptor protein
Npas2: Neuronal PAS domain protein 2
NPC1L1: NPC1-like intracellular cholesterol transporter 1
NRG1: Neuregulin 1
NTRK2: Neurotrophic tyrosine kinase receptor type 2
OCD: Obsessive-compulsive disorder
OCRDs: Obsessive-compulsive and related disorders
OCS: Obsessive-compulsive symptoms
OFC: Orbitofrontal cortex
OLIG2: Oligodendrocyte transcription factor 2
OR: Odds ratio
PANDA: Pediatric autoimmune neuropsychiatric disorders associated with streptococcal infections
PANS: Pediatric acute neuropsychiatric syndrome
Per: Period (part of the Per family)
PET: Positron emission tomography
PGRN: Progranulin
PI3K: Phosphoinositide 3-kinase
PIK3CG: Phosphatidylinositol-4,5-bisphosphate 3-kinase catalytic subunit gamma
PKC: Protein kinase C
PLC: Phospholipase C
PPARG: Peroxisome proliferator-activated receptor gamma
pPut: Posterior portion of putamen
PRKD3: Protein kinase D3
PRKD3: Protein kinase D3
PRP: Post-reproductive period
PRS: Polygenic risk score
Psma: Pre-supplementary motor area
PTBP2: Polypyrimidine tract binding protein 2
PTPN22: Protein tyrosine phosphatase non-receptor type 22
PTPRD: Protein tyrosine phosphatase receptor type D
PTSD: Post-traumatic stress disorder
Q-FISH: Quantitative fluorescent *in situ* hybridization
RFLP: Restriction fragment length polymorphism
RNA: Ribonucleic acid
Rora/Rorb/Rorc: Retinoic acid receptor-related orphan receptors A/B/C
RS: Reference SNP cluster ID
RT: Relaxation therapy
rTMS: Repetitive transcranial magnetic stimulation
SARS-CoV-2: Severe acute respiratory syndrome coronavirus 2
SCARB1: Scavenger receptor class B member 1
SCN1A: Sodium voltage-gated channel alpha subunit 1
SCNA: Alpha-synuclein
SCZ: Schizophrenia
SETD5: SET domain containing 5
SHARPIN: SHANK-associated RH domain-interacting protein
SKY: Spectral karyotyping
SLC: Solute carrier
SLC18A1: Solute carrier family 18 member A1
SLC1A1: Solute carrier family 1 member 1
SLC1A1: Solute carrier family 1 member 1 (glutamate transporter)
SLC30A8: Solute carrier family 30 member 8
SLC6A4: Solute carrier family 6 member 4 (a gene associated with serotonin transport)
SLITRK1: SLIT and NTRK-like family member 1
SLITRK5: SLIT And NTRK-like family member 5
SMA: Supplementary motor area
SMAD4: SMAD family member 4
sMRI: Structural magnetic resonance imaging
SNP: Single nucleotide polymorphism
SNr: Substantia nigra pars reticulata
SNRI: Serotonin-norepinephrine reuptake inhibitor
SPECT: Single-photon emission tomography
SRI: Serotonin reuptake inhibitor
SSCP: Single-strand conformation polymorphism
SSRI: Selective serotonin reuptake inhibitor
STAT4: Signal transducer and activator of transcription 4
STN: Subthalamic nucleus
STX1A: Syntaxin 1A
STXBP1: Syntaxin-binding protein 1
T1D: Type 1 diabetes
T2D: Type 2 diabetes
TALENs: Transcription activator-like effector nucleases
TCA: Tricarboxylic acid
TCA: Tricyclic antidepressant
TCF7L2: Transcription factor 7-like 2
TCGA: The Cancer Genome Atlas
tCGH: Translocation comparative genomic hybridization
Tef: Transcription factor/thyrotroph embryonic factor
TH: Tyrosine hydroxylase gene
THBS2: Thrombospondin-2
TNF-α: Tumor Necrosis Factor-Alpha
TOMM40: Translocase of outer mitochondrial membrane 40
TPH1: Tryptophan hydroxylase 1
TPH2: Tryptophan hydroxylase 2
UC: Ulcerative colitis
vCaud: Ventral part of the caudate nucleus
VGLUT: Vesicular glutamate transporter
vlPFC: Ventrolateral prefrontal cortex
vmPFc: Ventromedial prefrontal cortex
VNTR: Variable number of tandem repeats
WHO: World Health Organization
WWOX: WW domain containing oxidoreductase
Y-BOCS: Yale-Brown Obsessive-Compulsive Scale
